# Sacrificial Mechanical Bond is as Effective as a Sacrificial Covalent Bond in Increasing Cross-Linked Polymer Toughness

**DOI:** 10.1021/jacs.3c08595

**Published:** 2023-10-18

**Authors:** Hirogi Yokochi, Robert T. O’Neill, Takumi Abe, Daisuke Aoki, Roman Boulatov, Hideyuki Otsuka

**Affiliations:** † Department of Chemical Science and Engineering, Tokyo Institute of Technology, 2-12-1 Ookayama, Meguro-ku, Tokyo 152-8550, Japan; ‡ Department of Chemistry, University of Liverpool, Liverpool L69 7ZD, U.K.; § Department of Applied Chemistry and Biotechnology, Graduate School of Engineering, Chiba University, 1-33 Yayoi-cho, Inage-ku, Chiba-shi, Chiba 263-8522, Japan

## Abstract

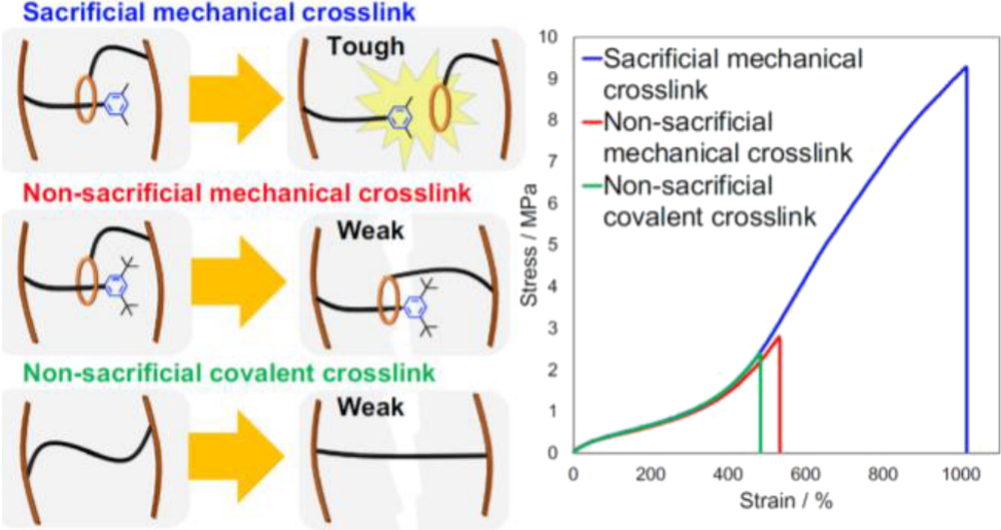

Sacrificial chemical bonds have been used effectively to increase the toughness of elastomers because such bonds dissociate at forces significantly below the fracture limit of the primary load-bearing bonds, thereby dissipating local stress. This approach owes much of its success to the ability to adjust the threshold force at which the sacrificial bonds fail at the desired rate, for example, by selecting either covalent or noncovalent sacrificial bonds. Here, we report experimental and computational evidence that a mechanical bond, responsible for the structural integrity of a rotaxane or a catenane, increases the elastomer’s fracture strain, stress, and energy as much as a covalent bond of comparable mechanochemical dissociation kinetics. We synthesized and studied 6 polyacrylates cross-linked by either difluorenylsuccinonitrile (DFSN), which is an established sacrificial mechanochromic moiety; a [2]rotaxane, whose stopper allows its wheel to dethread on the same subsecond time scale as DFSN dissociates when either is under tensile force of 1.5–2 nN; a structurally homologous [2]rotaxane with a much bulkier stopper that is stable at force >5.5 nN; similarly stoppered [3]rotaxanes containing DFSN in their axles; and a control polymer with aliphatic nonsacrificial cross-links. Our data suggest that mechanochemical dethreading of a rotaxane without failure of any covalent bonds may be an important, hitherto unrecognized, contributor to the toughness of some rotaxane-cross-linked polymers and that sacrificial mechanical bonds provide a mechanism to control material fracture behavior independently of the mechanochemical response of the covalent networks, due to their distinct relationships between structure and mechanochemical reactivity.

## Introduction

**Figure 1:**
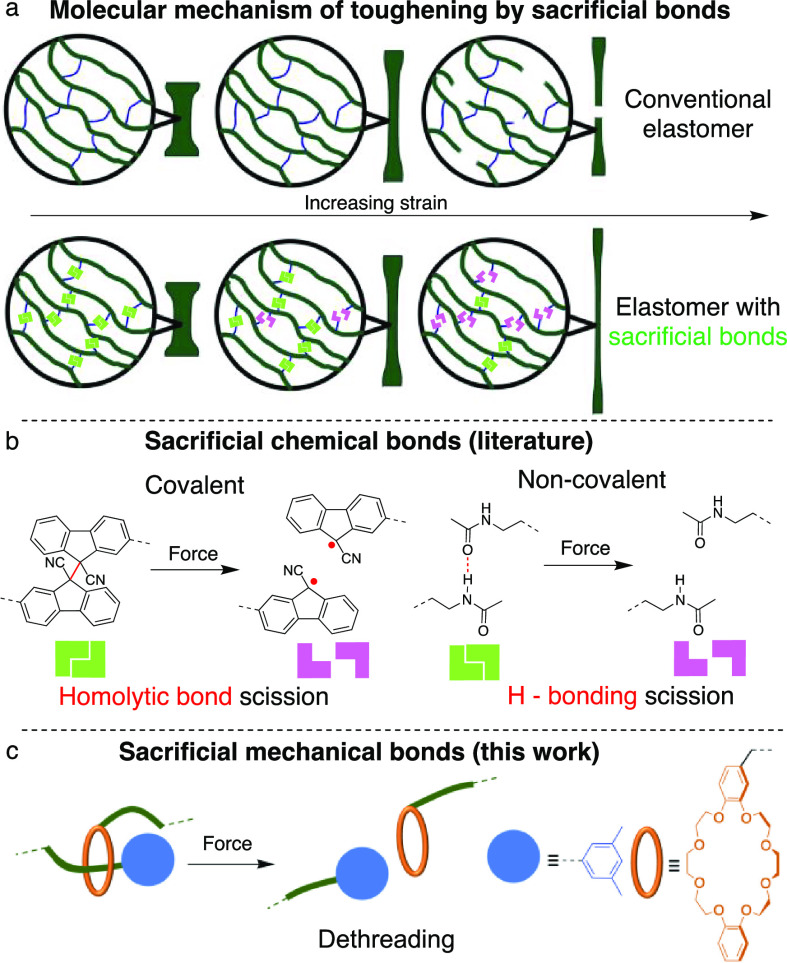
Sacrificial bonds in polymers. (a) Incorporating mechanochemically labile bonds into the molecular network of an elastomer increases its toughness because fracture of such sacrificial bonds at applied mechanical loads below the stability limit of the dominant network bond effectively dissipates local molecular strain, thereby increasing the amount of mechanical energy the material absorbs before failing. (b) Chemical covalent and noncovalent bond have been used extensively as sacrificial bonds. (c) A mechanical bond, in the form of a thermally stable and mechanochemically dethreadable rotaxane, increases the toughness of a polyacrylate elastomer as much as a well-studied covalent sacrificial bond, at comparable densities.

A common strategy to increase elastomer toughness is to incorporate sacrificial chemical bonds into its molecular network.^1−3^ Because sacrificial bonds are more dissociatively labile than the majority of the bonds comprising the network, their scission at moderate loads increases the amount of mechanical energy that the material can absorb without failing, i.e., they increase material toughness (Figure 1a).^4−6^ To date, this strategy was demonstrated with both noncovalent^7−9^ and covalent sacrificial bonds (Figure 1b).^1,10−12^ The former dissociates at low loads (single-chain forces <1 nN) and reform rapidly once the load dissipates.^7,13^ Sacrificial covalent bonds dissociate at higher loads (>1.5 nN) and most require energy input (usually in the form of heat or light) to reform,^14,15^ although several notable examples of spontaneous regeneration of covalent sacrificial bonds upon load dissipation have also been described.^16,17^ Sacrificial bonds whose dissociation either fractures a polymer backbone or releases hidden length have been used successfully to increase polymer toughness.^2,3,18,19^

A mechanical bond, that keeps the ring of a rotaxane threaded through its axle and maintains the two macrocycles of a catenane interlocked, is considered a distinct type of chemical interaction.^20−22^ Depending on the relative sizes of its wheel and the stopper, the mechanical bond of a rotaxane can be broken without affecting any of its covalent bonds.^23^ Such selective dethreading caused by a tensile force acting between the wheel and the axle was reported to occur in single-molecule force experiments^24^ and in stretched bulk polymers,^25^ but not in a sonicated solution.^26^ These observations suggest a possibility, hitherto unexplored, of using the mechanical bond as a sacrificial moiety for improving the fracture resistance of elastomers (Figure 1c).

Polymers incorporating (nonsacrificial) mechanical bonds are well established and have diverse molecular compositions, network topologies, and macroscopic properties.^26−35^ Such polymers are usually designed to prevent dethreading unless a covalent network fractures, and their unusual mechanical properties are thought to reflect the capacity of a mechanical bond to maintain a pair of polymer segments in point contact without impairing their ability to slide past each other in response to macroscopic mechanical load.^36^ However, the dynamic complexity arising from mechanically interlocked components means that our understanding of the molecular processes responsible for the properties of such polymers, including the relative contributions of sliding,^25,37−42^ dethreading^43^ and covalent bond fracture^18^ to dissipation of mechanical stress, remains limited.

**Figure 2:**
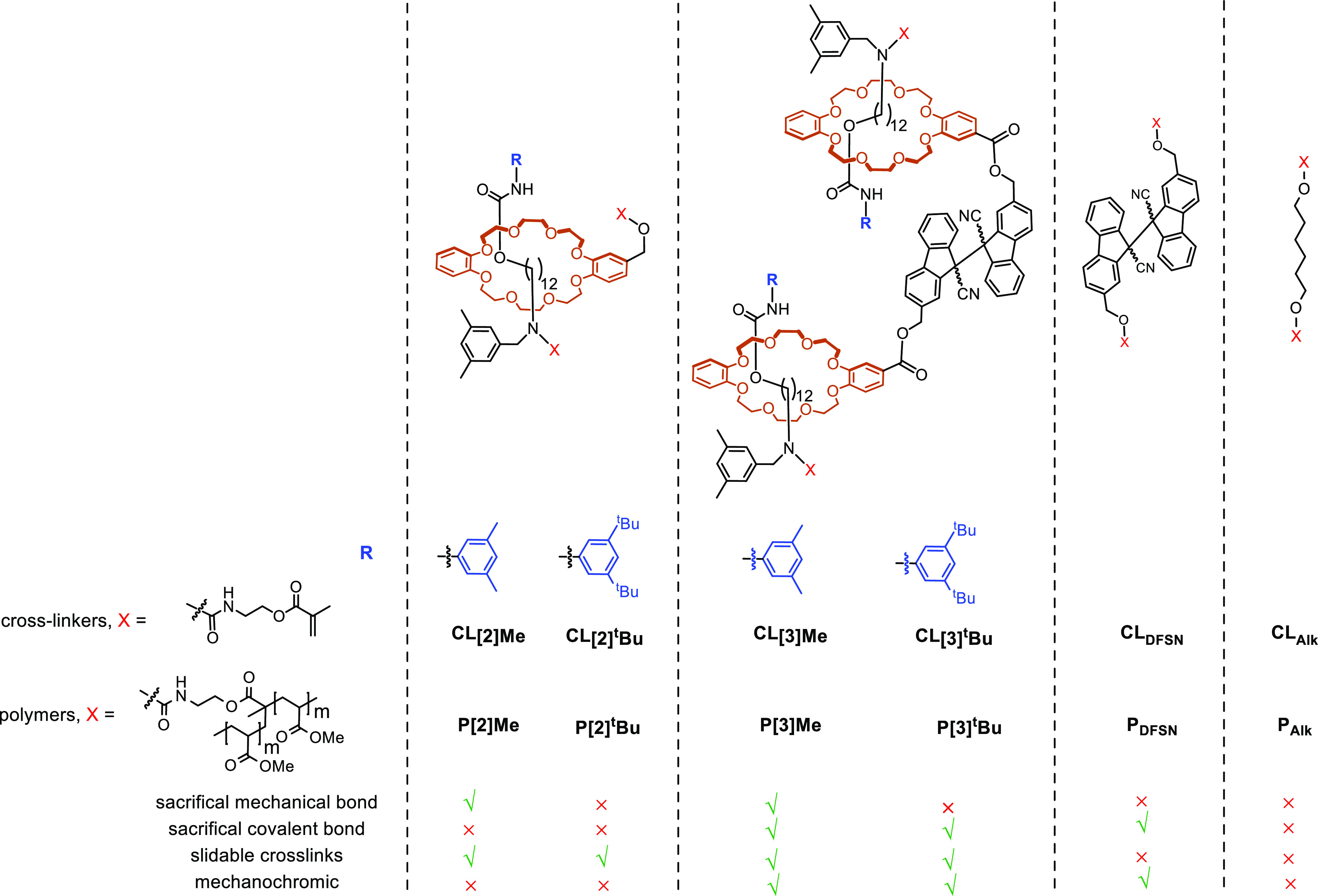
Structure and properties of the 6 polymers used in this study and their precursor cross-linkers. The position of DFSN in **CL[3]** cross-linkers ensures that dethreading does not require the wheel to traverse the DFSN moiety and consequently, the dethreading kinetics in **CL[3]** is expected to be comparable to that of **CL[2]** analogs. In the notation of rotaxane cross-linkers (and their polymers), [2] and [3] refer to the number of mechanically interlocked components, in compliance with the IUPAC nomenclature for such molecules, and Me and ^t^Bu identify the alkyl substituents of the aromatic stoppers.

Here, we report experimental and computational data, suggesting that a sacrificial mechanical bond is as effective at increasing the tensile toughness of an elastomer as a well-established sacrificial covalent bond. We synthesized and characterized the mechanical properties of 6 poly(methyl acrylate)s shown in Figure 2 whose cross-links contained only sacrificial mechanical bonds in the form of a dethreadable rotaxane, **P[2]Me**; only sacrificial covalent bonds of mechanochromic difluorenylsuccinonitrile (DFSN),^44^**P[3]**^**t**^**Bu** and **P**_**DFSN**_; both types of sacrificial bonds, **P[3]Me**; or neither (**P[2]**^**t**^**Bu**, and **P**_**Alk**_). Across this series, the mechanical sacrificial bond yielded material (**P[2]Me**) with the highest fracture energy, whereas both types of sacrificial bonds were comparably effective in increasing the material's fracture strain and stress, compared to analogs lacking sacrificial bonds. A comparison of materials containing both DFSN and a rotaxane (**P[3]Me** and **P[3]**^**t**^**Bu**) demonstrates that competition between failure of sacrificial mechanical and covalent bonds can be controlled predictably to favor either one or the other as the primary dissipative mechanism. Finally, our data suggest that even short cross-linker sliding distances of <3 nm measurably improve material failure properties.

We chose DFSN as the source of the covalent sacrificial bond because its dissociation is mechanochromic, facilitating detection, and has previously been demonstrated to effectively increase the toughness of elastomers.^45^ We implemented the mechanical bond in the form of rotaxanes of the general structure in Figure 2 because they are most commonly used in elastomers due to the ease of synthesis and incorporation into molecular networks.^38^ Because rotaxane cross-links may contribute to dissipation of mechanical load by chain sliding even in the absence of a sacrificial mechanical bond, we sought to compare properties of elastomers linked by two structurally related rotaxanes: one that is inert to dethreading at force >5 nN and the other whose dethreading kinetics at 1–2 nN is comparable to that of the reference covalent sacrificial bond.

## Results and Discussion

**Figure 3:**
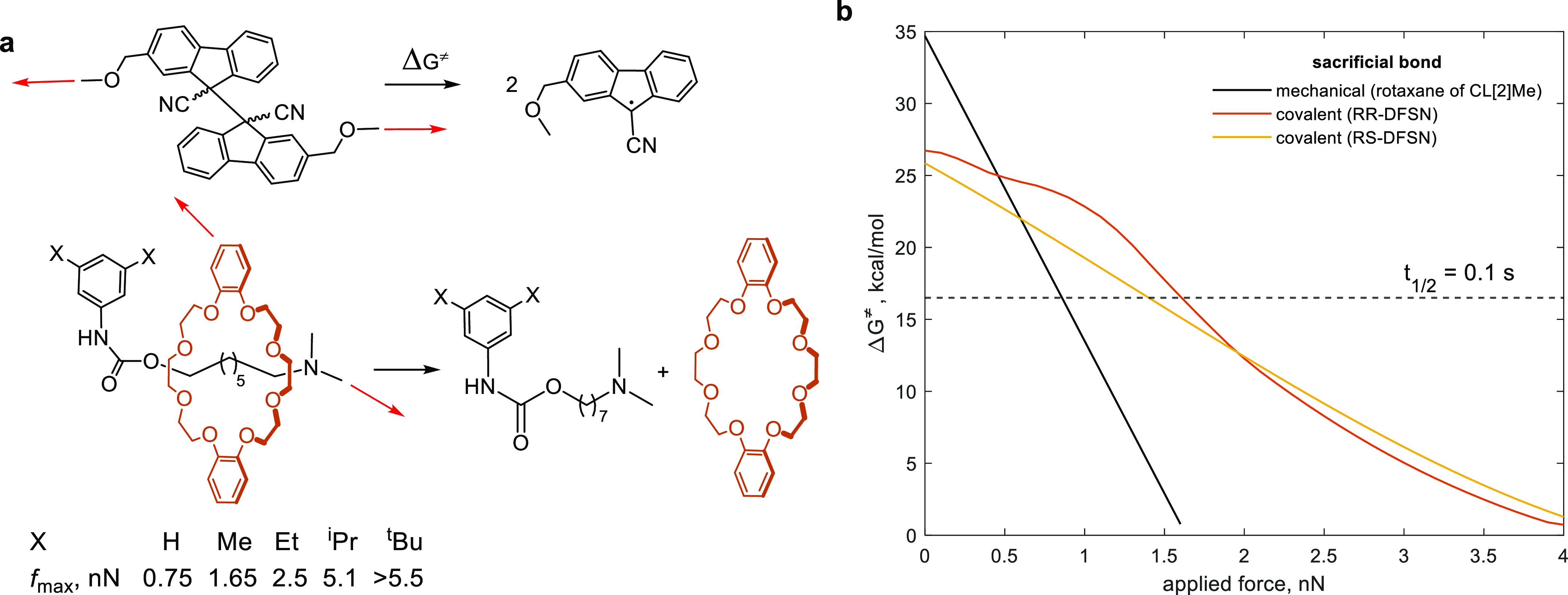
Summary of DFT computations. (a) Chemical reactions whose mechanochemical kinetics were computed; red arrows indicate the location and the direction of applied force vectors. (b) Force-dependent activation free energies, Δ*G*^‡^(*f*), for the sacrificial bonds studied experimentally. For reference, the horizontal line indicates the activation energy corresponding to half-life, *t*_1/2_, of 0.1 s, or the approximate time scale of reactions affecting stress/strain curves. The converged geometries of the rotaxanes closest to *f*_max_ and computed Δ*G*^⧺^(*f*) for DFSN dissociation are tabulated in Tables S2 and S3.

Although DFSN has been used extensively as a mechanosensitive moiety,^10,11,47^ its mechanochemical kinetics has not been reported. We calculated the activation free energy for dissociation of a DFSN derivative as a function of the tensile force applied at the C atoms of its methoxy groups (Figure 3). Because the C atoms of DFSN bound by the scissile bond are chiral, DFSN derivatives exist as diastereomers. Our calculations at the uBMK/6-31+G(d) level in vacuum suggest that both diastereomers are thermodynamically stable at room temperature (standard reaction free energies, Δ*G*_0_, are 13.9 and 13.6 kcal/mol, for dissociation of RR and RS isomers, respectively) and their dissociation kinetics is similarly moderately sensitive to extrinsic force of >0.2 nN (slopes of Δ*G*^‡^ vs *f* correlations are −6.7 and −5.2 kcal/mol/nN for RR and RS isomers). Calculated Δ*G*^‡^(*f*) suggests that mechanochemical dissociation of DFSN in a loaded elastomer is likely to occur at local force ≥1.5 nN. This threshold is significantly lower than that derived with COGEF, which adds to the increasing volume of evidence^62^ that the superficially appealing simplicity of COGEF requires aphysical assumptions that render its conclusions both conceptually and empirically suspect. Recombination of the product radicals upon dissipation of the local load is diffusion-limited in amorphous solids and melts,^48^ but is slower than the diffusion rate in solution (recombination Δ*G*_0_^‡^ = 13.6 and 12.5 kcal/mol), in accordance with the reported observations.^44,49^ Recombination likely produces approximately equimolar mixtures of the diastereomers. Calculations with the ethyl acetate-parametrized SMD model of the reaction medium at uBMK/6-31+G(d) or at uMPW1*K*/6-31+G(d) in vacuum yielded very similar conclusions (Figure S14).

The kinetics and mechanisms of rotaxane dethreading that occurs without concomitant rearrangement of covalent bonds has been little studied apart from qualitative observations about the importance of the size of the stopper group^24,46^ (blue in Figure 2). Consequently, we used DFT calculations to identify the stoppers (Figure 3) with potentially exploitable dethreading kinetics under a mechanical load. The large size of candidate rotaxanes and the likely complex, multistep dethreading mechanism that traverses multiple shallow energy minima typical of conformational transitions^50^ precluded optimizations of the transition states, and hence calculations of activation free energies, and required us to optimize the geometries at a less computationally demanding level of BLYP/6-31G(d).

To establish the limit of mechanical stability of candidate rotaxanes, we optimized each rotaxane coupled to a series of increasingly large stretching forces applied between the C of a terminal methyl group at one terminus of the model axle and the distal aromatic C atom of the wheel (red arrows, Figure 3a). For all but the largest ^t^Bu-substituted stopper, increasing the applied force above a threshold (*f*_max_ in Figure 3) caused the geometry optimization to converge to the wheel separated from the axle, i.e., force above *f*_max_ eliminates the energy minimum corresponding to the threaded geometry without breaking any covalent bonds.

These results identified the 3,5-dimethylphenyl stopper (rotaxanes **CL[2]Me** and **CL[3]Me**) as most likely to yield a mechanical sacrificial bond of comparable mechanochemical lability to the scissile C–C covalent bond of DFSN and the 3,5-di(*tert*-butyl)phenyl stopper to create nonsacrificial mechanical bond (**CL[2]**^**t**^**Bu**, **CL[3]**^**t**^**Bu**). We estimated Δ*G*^‡^(*f*) of the sacrificial mechanical bond by assuming that it decreases linearly with force (which has been previously demonstrated to be a reasonable approximation for diverse isomerization reactions^51,52^) from Δ*G*_0_^‡^ = 34.7 ± 0.2 kcal/mol at 0 nN to 0 kcal/mol at *f*_max_ = 1.65 nN. The applied force likely decreases the number of sequential conformational transitions needed to dethread, because force eliminates shallow conformational minima^53,54^ and also strains the remaining intermediates and transition states, and plausibly changes their relative energies.^55^ Such changes were previously demonstrated to be compatible with approximating the true force-dependent activation energies with linear dependences on the applied force at acceptable levels of accuracy.^56−58^

We obtained Δ*G*_0_^‡^ from monitoring a solution of a derivative of **CL[2]Me** in toluene-d_8_ by ^1^H NMR spectroscopy at 110 °C, which revealed detectable dethreading in 5 days without evidence of any side reactions (Figure S7). Under the same conditions, dethreading over the 3,5-di(*tert*-butyl)phenyl stopper in **CL[2]**^**t**^**Bu** was undetectable (Figure S8), consistent with its much larger calculated *f*_max_. Because dethreading is a unimolecular reaction, its Δ*G*^‡^ is likely only weakly temperature-dependent, justifying our use of the energy at 110 °C to estimate room-temperature mechanochemical kinetics.

**Figure 4:**
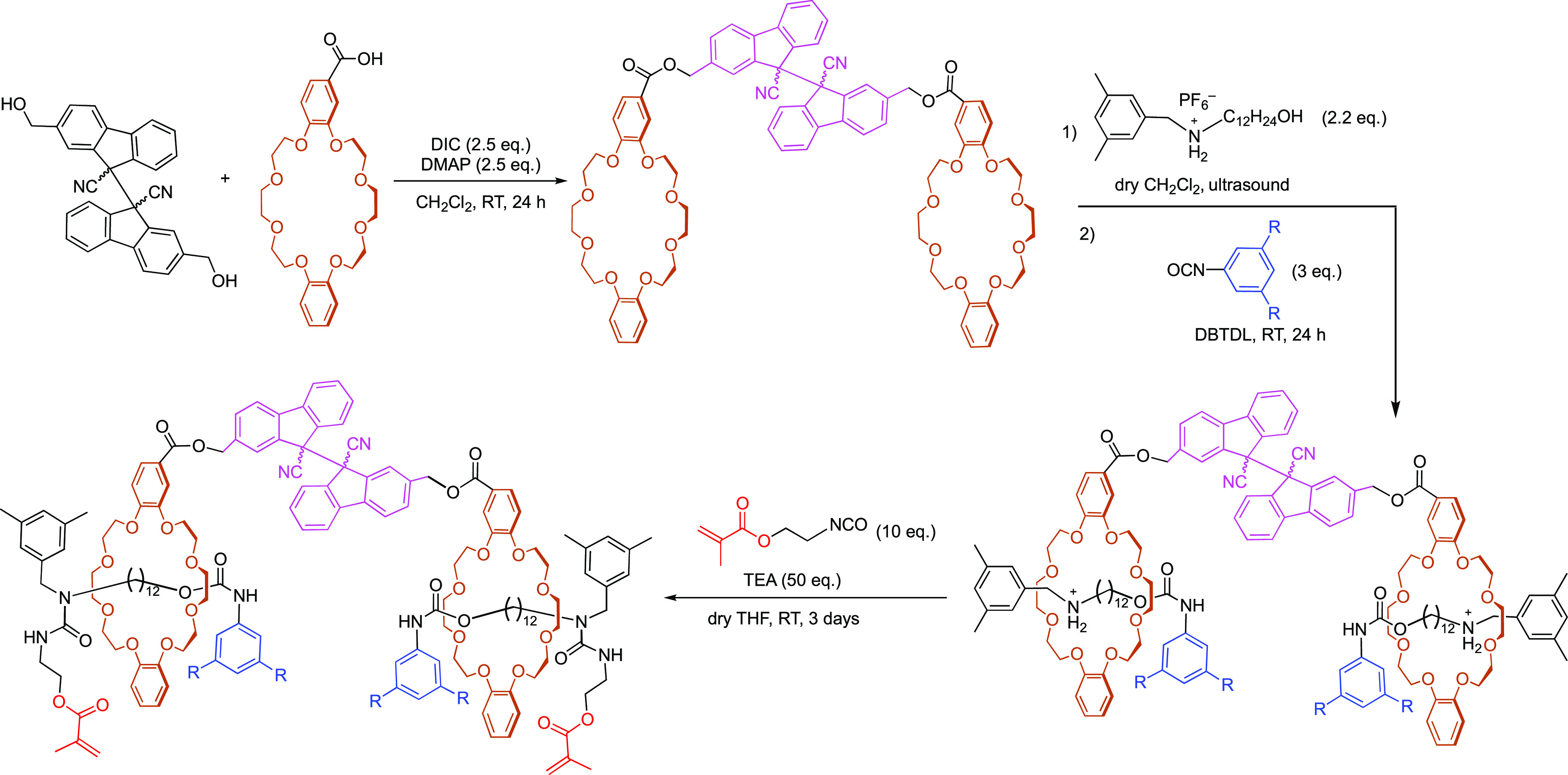
Synthesis of [3]rotaxane cross-linkers **CL[3]Me** (R = Me) and **CL[3]**^**t**^**Bu** (R = ^t^Bu). DBTDL = dibutyltin dilaurate, and TEA = triethylamine. See Figures S5 and S6 for spectroscopic characterization.

We synthesized bis-methacrylate cross-linkers, **CL[2]Me**,^41^**CL[2]**^**t**^**Bu**,^59^**CL**_**DFSN,**_^11^ and **CL**_**Alk**_(11) (Figure 2) according to literature procedures. [3]Rotaxane bisacrylates **CL[3]Me** and **CL[3]**^**t**^**Bu** are new and were synthesized in 3 steps and >70% overall isolated yield (Figure 4) from described precursors (see the Supporting Information for further details). The products were characterized by ^1^H NMR spectroscopy and HR-ESI-TOF mass spectrometry (Figures S5–S8). We prepared the elastomers by room-temperature radical polymerization of methyl acrylate containing 1% mol cross-linker initiated with 2,2′-Azobis(4-methoxy-2,4-dimethylvaleronitrile) over 2 days, followed by repeated swelling and washing of the resulting material in CHCl_3_ and MeOH before drying. We chose PMA for these proof-of-the-concept experiments because its *T*_g_ of 10–20 °C makes it suitable for tensile testing under our conditions while retaining sufficient viscosity to prevent rapid recombination of FSN radicals generated by mechanochemical dissociation of DFSN, thus enabling mechanochromic identification of sacrificial covalent bond fracture.^11^ All polymers had comparable *T*_g_ of ∼15–20 °C and **P[2]Me**, **P[2]**^**t**^**Bu**, **P**_**DFSN**_ and **P**_**Alk**_ had swelling degrees (*Q*) within 7% of each other, suggesting negligible impact of the stopper group or presence of a mechanical bond on the network structure. As speculated previously,^60^ the 40% larger *Q* values of **P[3]Me-P[3]**^**t**^**Bu** compared to **P[2]Me**-**P[2]**^**t**^**Bu** may be attributable to the 2-fold difference in the respective ranges of available sliding motion (3.2 nm vs 1.6 nm).

**Figure 5:**
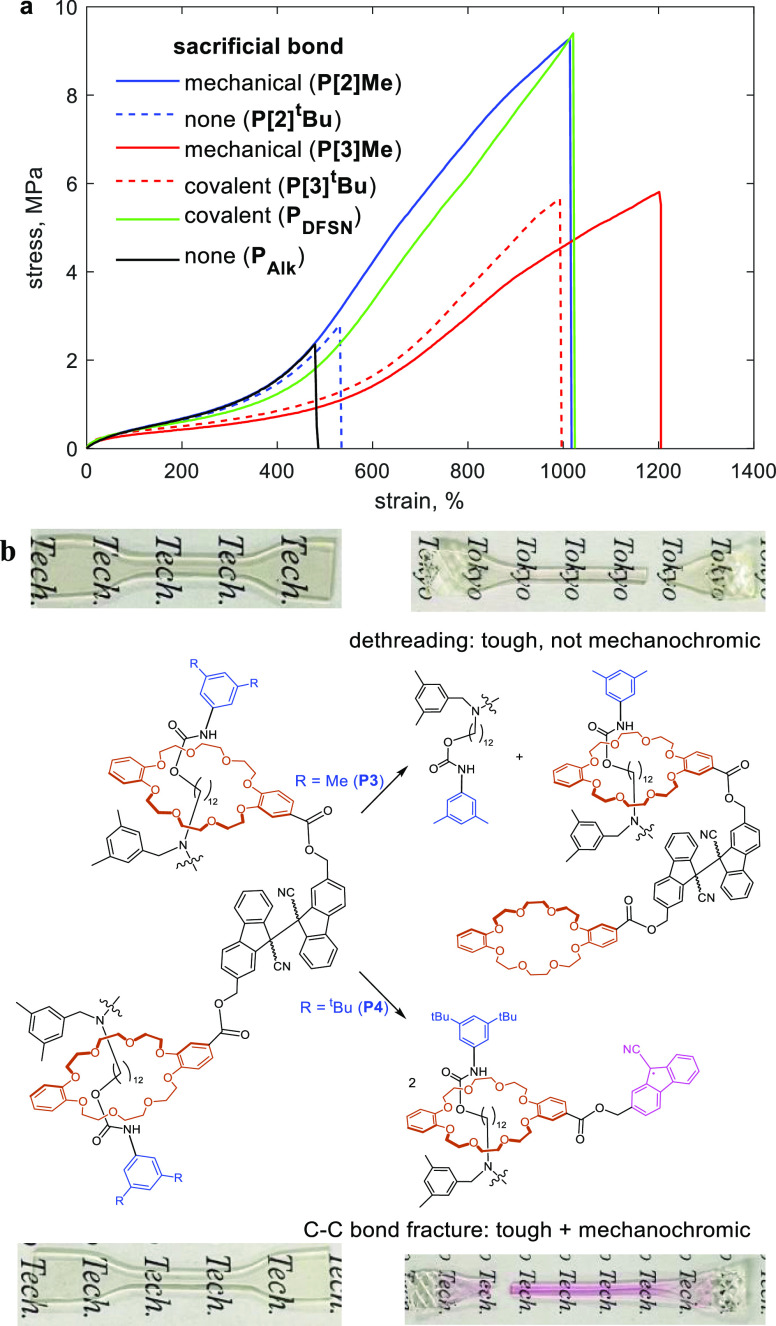
Summary of bulk mechanical properties of all studied polymers. (a) Stress/strain curves, each averaged over 3 independent measurements. Blue and red curves are for acrylates cross-linked with either [2]rotaxane or [3]rotaxane, respectively. (b) Intact and fractured samples of elastomers cross-linked with [3]rotaxanes containing mechanochromic DFSN in their axles and the mechanochemical reactions plausibly contributing to material failure.

We characterized the mechanical properties of the samples by measuring the stress/strain curves (Figures 5a and S11) up to failure of ∼0.7 mm thick dog-bone shaped samples at 40 °C and strain rate of 10 mm/min. The key measured parameters are summarized in Table 1. The presence of sacrificial bonds, whether covalent (**P[3]**^**t**^**Bu**, **P**_**DFSN**_) or mechanical (**P[2]Me**, **P[3]Me**) more than doubles the fracture strain and increases the fracture stress and fracture energy 2–4 fold and 3–9 fold, respectively, compared to polymers lacking sacrificial bonds (**P[2]**^**t**^**Bu** and **P**_**Alk**_). Pink coloration of loaded samples of **P[3]**^**t**^**Bu** (Figure 5b) or **P**_**DFSN**_ (Figure S10) suggests mechanochemical load-induced dissociation of DFSN, which produces chromophores and is thought to be the primary mechanism of dissipating mechanical stress in loaded elastomers containing DFSN cross-links.^11^ The comparable stress/strain curves of **P[2]Me** (mechanical sacrificial bond) and **P**_**DFSN**_ (covalent sacrificial bond) demonstrate that the sacrificial mechanical bond is as effective as a conventional sacrificial covalent bond at improving fracture limits of the elastomer.

**Table 1:** Key Parameters of Stress-Strain Behavior of All Studied Elastomers

sample	sacrificial bond	mechanochromic moiety?	slidable cross-links?	Young’s modulus, MPa^a^	fracture strain, %	fracture stress, MPa	fracture energy density, MJ/m^3^
**P[2]Me**	mechanical	N	Y	0.10 ± 0.06	1070 ± 65	9.20 ± 0.94	40.9 ± 8.2
**P[2]^t^Bu**	none	N	Y	0.99 ± 0.03	535 ± 14	2.68 ± 0.16	5.51 ± 0.39
**P[3]Me**	mechanical + covalent	Y	Y	0.98 ± 0.04	1250 ± 99	6.10 ± 0.65	29.3 ± 6.4
**P[3]^t^Bu**	covalent	Y	Y	1.4 ± 0.04	980 ± 78	5.54 ± 0.78	18.1 ± 4.2
**P_DFSN_**	covalent	Y	N	1.2 ± 0.1	1030 ± 41	9.17 ± 0.24	33.1 ± 2.5
**P_Alk_**	none	N	N	0.82 ± 0.01	486 ± 6	2.41 ± 0.11	4.51 ± 0.12

a Average stress/strain ratio at strain of 0–10%.

The lack of mechanochromism in **P[3]Me** (Figure 5b), which contains 2 sacrificial mechanical bonds per 1 sacrificial covalent bond is broadly consistent with dethreading being faster than C–C homolysis at single-chain forces >1.5 nN (Figure 3), further suggesting that the rotaxane in **P[3]Me** acts as a mechanochemical protective group of DFSN. In other words, the rotaxanes structure of **P[3]Me**, previously considered to be stable under mechanical force^38,59^ is so labile that its dethreading prevents the DFSN moiety from ever experiencing enough force to dissociate on the time scale of our experiments. Note that it is implausible for a single **CL[3]Me** cross-linker to both dethread and undergo DFSN dissociation. Either reaction places the remaining sacrificial moiety (i.e., rotaxanes in case of DFSN dissociation or DFSN in case of dethreading) at a terminus of a dangling chain, where it experiences no force and is therefore inert. The larger fracture strain of **P[3]Me** compared to **P[2]Me** (1250 ± 99% vs 1070 ± 65%) and the lower fracture stress in **P[3]**^**t**^**Bu** compared to **P**_**DFSN**_ (5.54 ± 0.78 vs 9.17 ± 0.24 MPa) probably reflect the longer range of accessible sliding motion in [3]rotaxane cross-links **CL[3]Me** and **CL[3]**^**t**^**Bu** compared to [2]rotaxane **CL[2]Me** and **CL[2]**^**t**^**Bu**. Similarly, the statistically significant 20% higher fracture energy of **P[2]**^**t**^**Bu** compared to **P**_**Alk**_, neither of which contains sacrificial bonds, may result from the capacity of rotaxane-based cross-links of **P[2]**^**t**^**Bu** to slide by up to 1.6 nm (the axle length).

## Conclusions

Our work illustrates that dissociation of a mechanical bond, realized here as a mechanochemically dethreadable rotaxane, comprises an effective energy dissipation mechanism that increases the toughness of an elastomer as much as a commonly used covalent sacrificial bond. Our findings suggest that dethreading may contribute to, or even dominate, the impressive toughness of elastomers cross-linked with 3,5-dimethylphenyl-stoppered rotaxanes,^38^ which hitherto has not been considered. More broadly, the reported results justify continued effort to identify practical implementations of sacrificial mechanical bonds, and the application niches in which such bonds may offer unique advantages, including in fundamental studies of load relaxation across polymer networks,^18,61^ mechanochemical feedback loops,^48^ and single molecule information storage.^63^ For example, our results suggest that the maximum tensile force a rotaxane can withstand without dethreading depends systematically on the steric bulk of the stoppers, potentially enabling the design of rich dynamic behavior resulting from kinetic competition between load relaxation by dethreading and the dissociation of covalent bonds. Such competition has been shown to allow detailed characterization of chain dynamics that is too complex for any alternative method^64^ and was previously speculated to enable detailed mechanistic studies and the design of new mechanoresponsive polymers.^18,48^ Unlike chemical sacrificial bonds, whose capacity to affect bulk^2,3,12,65,66^ and single-chain^67−70^ mechanical properties of polymers has been extensively studied and exploited for several decades now by polymer scientists, biophysicists, and physical chemists, the potential of a mechanical sacrificial bond remains wholly unexplored.

## Supporting Information Available

Syntheses of monomers and their precursors with details of their spectroscopic characterization, including images of ^1^H NMR and HR-MS spectra; ^1^H NMR data for kinetics of dethreading; polymerization procedures; details of characterization of the polymers; procedures for measuring stress/strain curves; details of DFT calculations; computed force-dependent free-energies of dissociation of DFSN; and Cartesian coordinates of the converged minimum-energy conformers of the rotaxanes coupled to force just below the stability threshold (PDF)
