# Supplementary material for: Sacrificial Mechanical Bond is as Effective as a Sacrificial Covalent Bond in Increasing Cross-Linked Polymer Toughness
Source: J Am Chem Soc. 2023 Oct 18;145(43):23794–801. doi: 10.1021/jacs.3c08595 (PMC10623562; doi:10.1021/jacs.3c08595)
Supplement: Supplementary file 1 — ja3c08595_si_001.pdf [file ja3c08595_si_001.pdf]

Supporting Information

A Sacrificial Mechanical Bond is as Effective as a Sacrificial Covalent Bond in Increasing Cross-Linked Polymers Toughness

Hirogi Yokochi, ≠a Robert T. O'Neill, ≠b Takumi Abe, a Daisuke Aoki *c Roman Boulatov *b and Hideyuki Otsuka *a

(a) Department of Chemical Science and Engineering, Tokyo Institute of Technology, 2-12-1 Ookayama, Meguro-ku, Tokyo 152-8550, Japan. (b) Department of Chemistry, University of Liverpool, Liverpool L69 7ZD, UK. (c) Department of Applied Chemistry and Biotechnology, Graduate School of Engineering, Chiba University, 1-33 Yayoi-cho, Inage-ku, Chiba-shi, Chiba 263-8522, Japan.

≠ Equal contribution

Corresponding Authors

* D. Aoki. Email: daoki@chiba-u.jp

* Roman Boulatov: R.Boulatov@liverpool.ac.uk

* H. Otsuka. Email: otsuka@polymer.titech.ac.jp

1. General information

All reagents and solvents were purchased from Sigma-Aldrich, Wako Pure Chemical Corporation, Tokyo Chemical Industry, and Kanto Chemical, and used as received, unless otherwise noted. **DFSN-diol**,[1] **DFSN-C**,[2] **CC**,[2] **DBC$_{24}$O$_8$-acrylate**,[3] **DBC$_{24}$O$_8$-COOH**,[4] **PA-axle** (phosphonium ammonium axle),[5] 3,5-di-tert-buthylphenyl isocyanate[6] and **CL[2]Me**,[7] were synthesized according to the cited methods. Methyl acrylate (MA) monomer was passed through an ammonia column to remove the inhibitor.

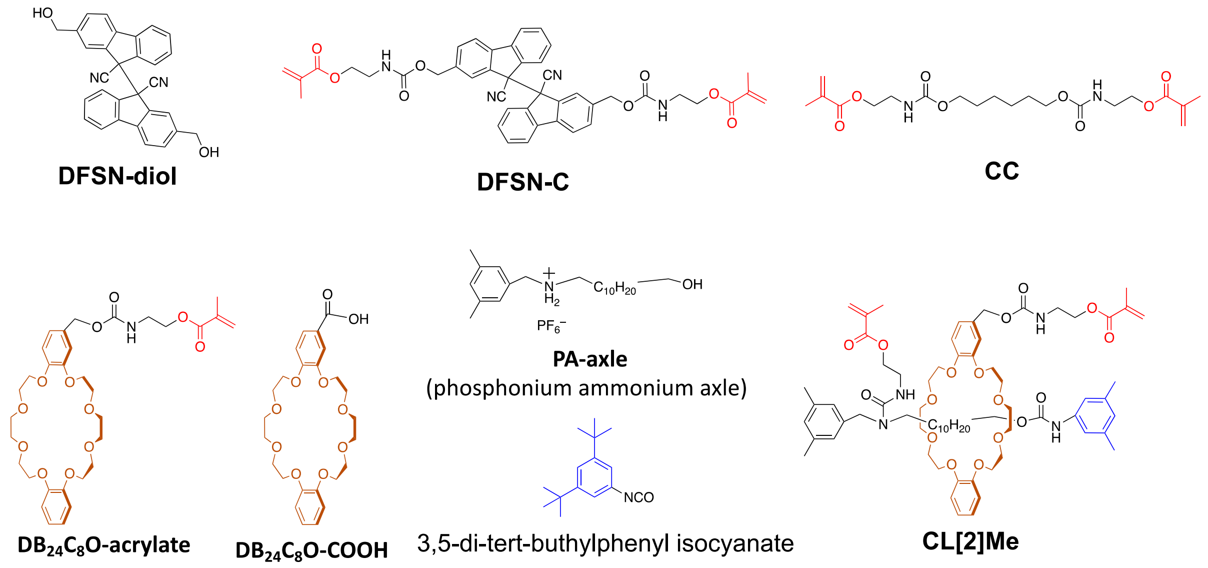

^{1}H spectra were recorded on a Bruker AVANCE III HD500 spectrometer with tetramethylsilane (TMS) as an internal standard in chloroform-d (CDCl$_3$) and toluene-*δ8*. ^{13}C NMR spectra were recorded on a JOEL JNM-ECZ400S/L1 spectrometer with tetramethylsilane (TMS) as an internal standard in chloroform-d (CDCl$_3$). Electrospray ionization mass spectrometry (ESI-TOF-MS) measurements were carried out on Bruker micrOTOF II. Preparative GPC were carried out on JAI LaboACE LC-5060 equipped with two gel permeation chromatography columns (JAIGEL-2HR Plus). Tensile tests were performed on a SHIMADZU AGS-X equipped with a thermostatic chamber (TCE-N300) and a 1 kN load cell at 40 °C. The films were punched out into dog-bone-shaped pieces standardized as JIS-7 (12 mm × 2 mm gauge section) with a thickness of 0.65–0.71 mm. These samples were stretched at a strain velocity of 10 mm/min. To evaluate the mechanical performance in each test, they were conducted more than three times and the average values were calculated together with their standard deviation. DSC measurements were carried out using a SHIMADZU DSC-60A Plus with a heating rate of 10 °C/min.

2. Synthesis

2.1. General procedure for [2]rotaxane synthesis

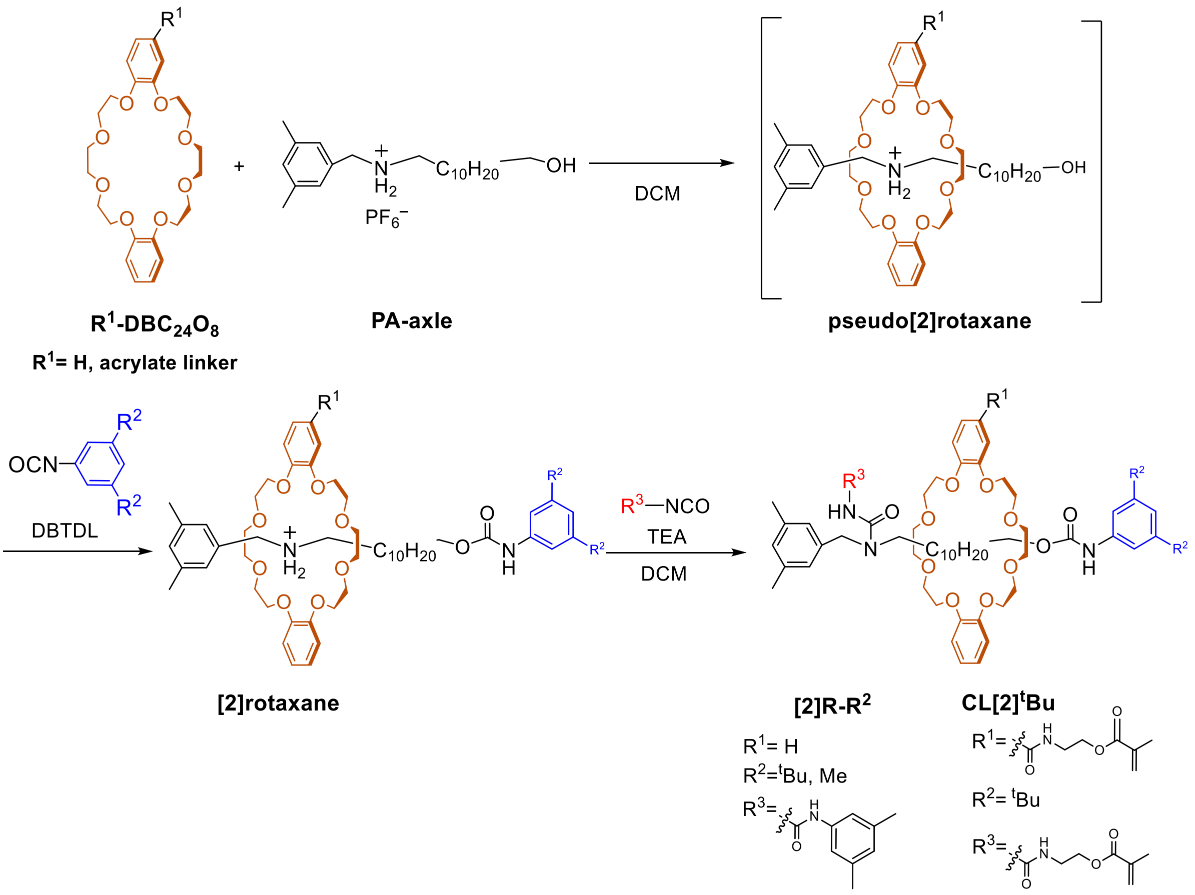

Scheme S1. Synthesis of [2]rotaxanes.

A mixture of **PA-axle** (1.1 equiv.) and **$DBC_{24}O_8$ - R^1** (1 equiv.) in dry DCM (1.00 – 4.00 mL) was sonicated at room temperature, until the solution became transparent indicating formation of pseudo[2]rotaxane. To a stock solution of the pseudo[2]rotaxane was added a few drops of dibutyltin dilaurate (DBTDL) and **3,5-R^2-phenyl isocyanate** (3.0 equiv)), before stirring for 24 hours to obtain crude [2]rotaxane. The mixture was quenched with ethanol and the solvent removed under reduced pressure. The resulting crude residue was diluted with dry THF (3.00 mL) and triethylamine (50 equiv.) and **R^3-NCO** (10 equiv.) was added in this order and stirred for 3 days at rt, after which the crude mixtures was purified by preparative GPC eluting with $CHCl_3$ to give the rotaxanes below.

[2]R-Me, R^1 = H, **R^2 = Me, R^3 =** 3,5-di-methylphenyl isocyanate

(42.0 mg, 39.6 µmol, 35.4%).

^{1}H-NMR (500MHz, $CDCl_3$, 298K): δ 8.28 (br, 1H), 7.16 (s, 2H), 6.94–6.82 (m, 13H), 6.63 (s, 1H), 6.51 (s,

1H), 6.28 (s, 1H), 4.48 (s, 2H), 4.30 (t, 2H), 4.19-4.07 (m, 8H), 3.91-3.79 (m, 8H), 3.56-3.47 (m, 8H), 3.37 (t, 2H), 2.30 (s, 6H), 2.24 (s, 6H), 2.10 (s, 6H), 1.79–0.87 (m, 20H) ppm; ^{13}C-NMR (400 MHz, 298K): δ 155.62, 154.17, 148.54, 139.72, 139.20, 138.63, 138.50, 138.12, 137.61, 129.33, 124.73, 124.58, 123.43, 120.76, 117.47, 115.97, 112.04, 69.82, 69.73, 68.09, 64.90, 50.70, 48.21, 31.68, 29.77, 29.74, 29.68, 29.60, 29.56, 29.05, 28.45, 27.13, 25.84, 22.75, 21.45, 21.42, 21.36, 14.22 ppm; ESI-TOF-MS (m/z): calcd for $[M+Na]^+$, $C_{63}H_{87}N_3O_{11}Na$, 1084.6238; found, 1084.6215.

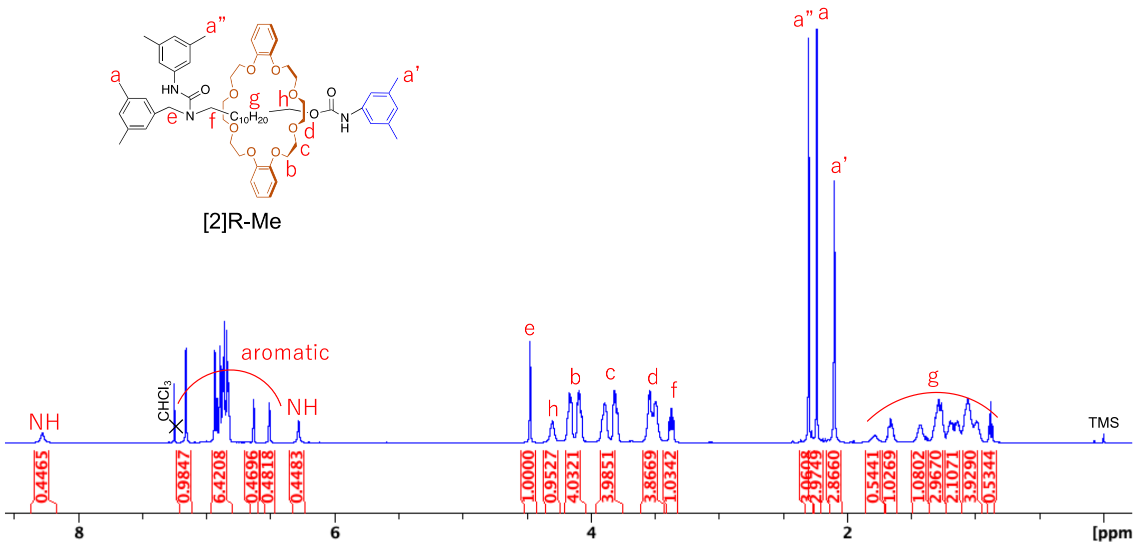

Figure S1. ^{1}H NMR spectrum of [2]rotaxane **[2]R-Me**. (500 MHz, 25 ºC, $CDCl_3$).

[2]R-^{t}Bu, **R^1**= H, **R^2 = ^{t}Bu**, **R^3=** 3,5-di-methylphenyl isocyanate

(49.5 mg, 42.9 µmol, 38.3%).

^{1}H-NMR (500MHz, $CDCl_3$, 298K): δ 8.05 (br, 1H), 7.27 (s, 2H), 6.97 (s, 3H), 6.89–6.81 (m, 11H), 6.62 (s, 1H), 6.51 (s, 1H), 6.45 (s, 1H), 4.48 (s, 2H), 4.39 (t, 2H), 4.19-4.10 (m, 8H), 3.96-3.77 (m, 8H), 3.65-3.55 (m, 8H), 3.39 (t, 2H), 2.28 (s, 6H), 2.22 (s, 6H), 1.72–0.87 (m, 38H) ppm; ^{13}C-NMR (400 MHz, 298K): δ 155.72, 154.38, 151.07, 148.57, 139.47, 139.19, 138.40, 137.97, 129.10, 124.79, 124.39, 120.88, 117.51, 115.81, 112.87, 112.40, 70.18, 69.86, 68.32, 65.12, 50.41, 48.09, 34.88, 31.69, 31.49, 29.86, 29.76, 29.69, 29.65, 29.11, 28.32, 27.05, 25.88, 22.76, 21.47, 21.44, 14.23 ppm; ESI-TOF-MS (m/z): calcd for $[M+Na]^+$, $C_{69}H_{99}N_3O_{11}Na$, 1168.7177; found, 1168.7156.

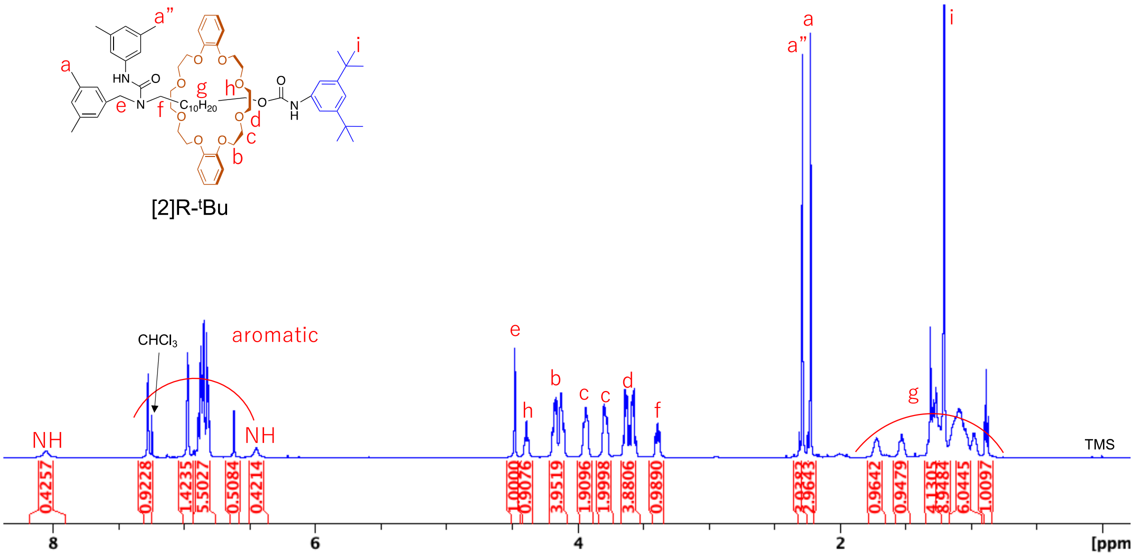

Figure S2. ^{1}H NMR spectrum of [2]rotaxane **[2]R-^{t}Bu**. (500 MHz, 25 ºC, $CDCl_3$).

CL[2]^{t}Bu, R^1= 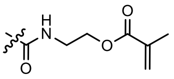 **, R^2 = ^{t}Bu, R^3=** 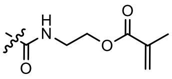 , (286 mg, 0.24 mmol, 90.1%).

^{1}H-NMR (500MHz, $CDCl_3$, 298K): δ 8.17 (br, 1H), 6.94–6.77 (m, 12H), 6.50 (s, 1H), 6.10 (s, 1H), 5.97 (s, 1H), 5.57 (m, 1H), 5.51 (m, 1H), 5.02 (br, 1H), 5.00 (s, 2H), 4.75 (br, 1H), 4.41 (t, 2H), 4.37 (s, 2H), 4.26–3.47 (32H), 3.25 (t, 2H), 2.27 (s, 6H), 1.93 (s, 3H), 1.86 (s, 3H), 1.70–0.90 (m, 38H) ppm; ^{13}C-NMR (400 MHz, 298K): δ 167.44, 167.30, 158.13, 156.45, 154.35, 150.95, 148.71, 148.61, 148.57, 139.37, 138.33, 138.02, 136.10, 136.00, 128.98, 128.73, 126.14, 125.79, 124.58, 121.20, 120.90, 115.59, 112.89, 112.77, 112.44, 112.11, 70.12, 69.87, 69.80, 68.48, 68.30, 67.09, 65.06, 64.31, 63.83, 50.25, 47.76, 40.32, 40.16, 34.87, 31.50, 29.85, 29.79, 29.71, 29.64, 29.13, 28.34, 27.10, 25.92, 21.42, 18.42, 18.35 ppm; ESI-TOF-MS (m/z): calcd for $[M+Na]^+$, $C_{75}H_{110}N_4O_{17}Na$, 1361.7764; found, 1361.7734.

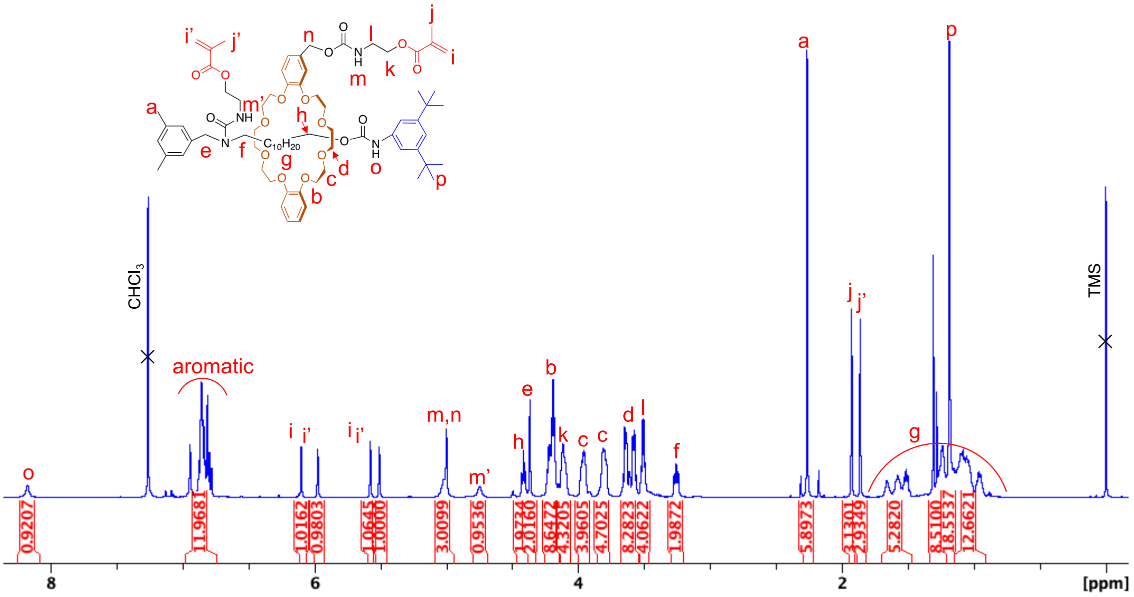

Figure S3. ^{1}H NMR spectrum of **CL[2]^{t}Bu** (500 MHz, 25 ºC, $CDCl_3$).

2.4. Synthesis of DFSN-wheel

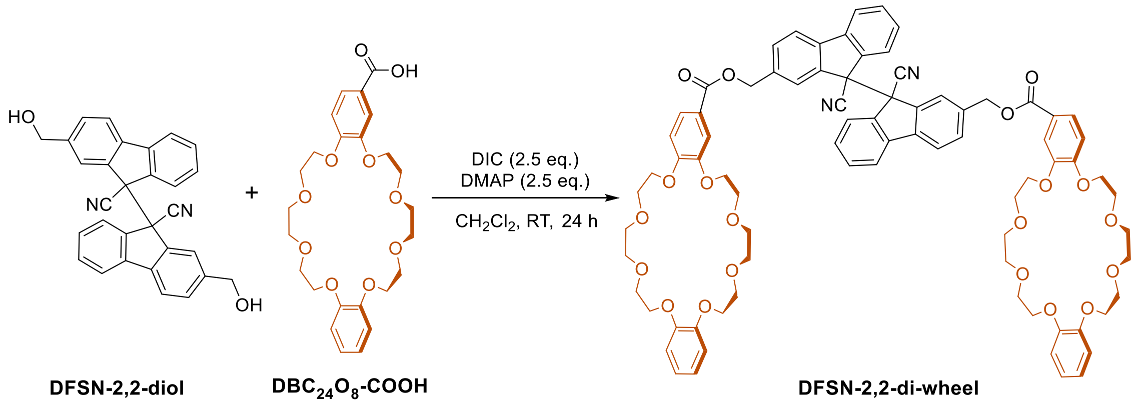

DFSN-2,2-diol **DBC$_{24}$O$_8$-COOH** **DFSN-2,2-di-wheel**

Scheme S4. Synthesis of rotaxane cross-linker **DFSN-2,2-di-wheel**.

DFSN-2,2-diol (500 mg, 1.14 mmol) and **DBC$_{24}$O$_8$-COOH** (1.40 g, 2.84 mmol) and DMAP (347 mg, 2.84 mmol) were dissolved in DCM (10.0 mL) and the mixture was stirred for 15 min at room temperature. Then N,N'-diisopropylcarbodiimide (437 μL, 2.84 mmol) was added to this solution at 0 °C and the mixture was stirred for 24 h at room temperature. The resulting solution was filtered and further purification was carried out by flash column chromatography with chloroform/methanol mixture (9/1, v/v) to afford **DFSN-2,2-di-wheel** as a white solid (1.02 g, 64.7%).

^{1}H-NMR (500MHz, $CDCl_3$, 298K): δ 7.72–6.83 (m, 14H, aromatic), 5.19 (br, 2H), 4.23-4.11 (br, 8H), 3.97-3.88 (br, 8H), 3.86-3.79 (br, 8H) ppm; ^{13}C-NMR (400 MHz, 298K): δ 165.89, 153.28, 148.96,

148.41, 140.80, 138.12, 136.46, 130.70, 130.38, 128.28, 125.30, 124.56, 124.32, 122.54, 121.50, 120.24, 118.36, 114.62, 114.50, 114.11, 112.08, 71.55, 71.42, 71.36, 70.02, 69.85, 69.69, 69.48, 69.41, 65.92, 53.68, 29.81, 22.46, 21.09 ppm; ESI-TOF-MS (m/z): calcd for $[M+Na]^+$, $C_{80}H_{80}N_2O_{20}Na$, 1411.5202; found, 1411.5190.

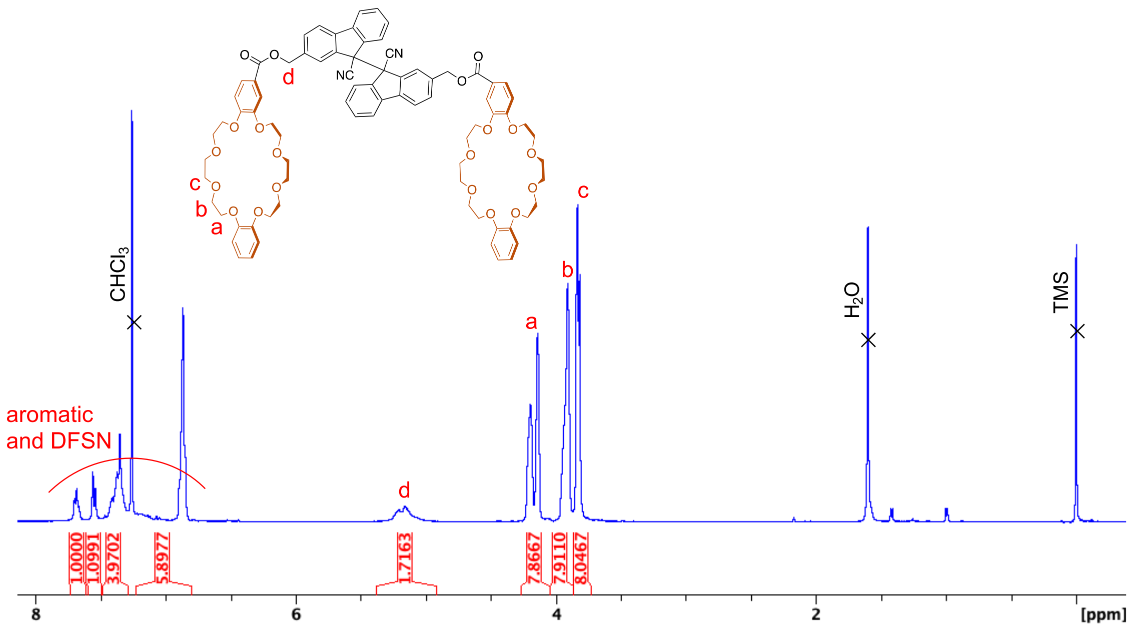

Figure S4. ^{1}H NMR spectrum of **DFSN-2,2-di-wheel** (500 MHz, 25 ºC, $CDCl_3$).

2.5. Synthesis of DFSN-containing [3]rotaxane cross-linker

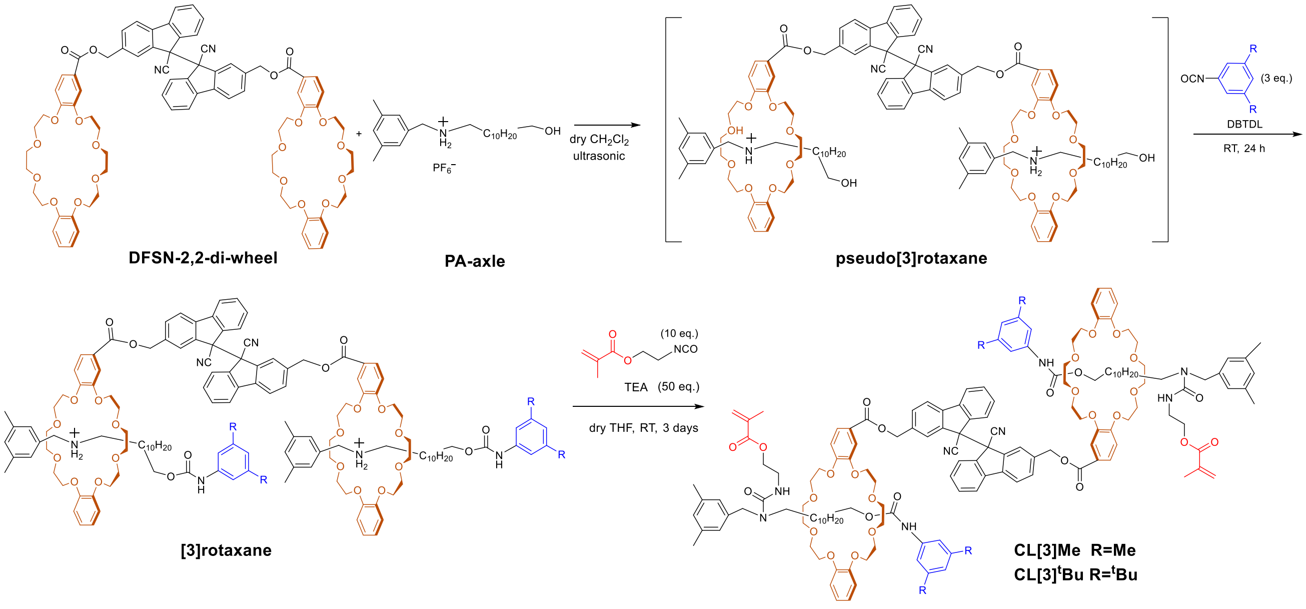

Scheme S5. Synthesis of rotaxane cross-linker DFSN-containing [3]rotaxane cross-linker.

A mixture of **PA-axle** (2.2 equiv.) and **DFSN-2,2-di-wheel** (1 equiv.) in dry DCM (4.00 mL) was sonicated at room temperature, until the solution became transparent indicating the formation of a pseudo[3]rotaxane. To a stock solution of the [3]rotaxane was added a few drops of dibutyltin dilaurate (DBTDL) and **R^2-NCO** (10 equiv.), and stirred for 24 hours to obtain the crude [3]rotaxane. The mixture was quenched with ethanol and the solvent removed under reduced pressure before dissolving the residue in dry THF (3.00 mL). To a solution of the crude [3]rotaxane in THF was added triethylamine (50 equiv.) and 2-isocyanate ethylmethacrylate (10 equiv.) in this order and stirred for 3 days at r.t. The crude was purified by preparative GPC eluting with $CHCl_3$ to give DFSN-containing [3]rotaxane cross-linker.

CL[3]Me (270 mg, 103 μmol, 71.3%), **R = Me**

^{1}H-NMR (500MHz, $CDCl_3$, 298K): δ 8.30 (br, 1H), 7.71–6.48 (m, 20H), 5.96 (s, 1H), 5.50 (m, 1H), 5.19 (br, 2H), 4.69 (br, 1H), 4.37 (s, 2H), 4.30–3.44 (30H), 3.25 (t, 2H), 2.27 (s, 6H), 2.07 (s, 6H), 1.85 (s, 3H), 1.55–0.98 (m, 20H) ppm; ^{13}C-NMR (400 MHz, 298K): δ 167.44, 166.02, 158.09, 154.10, 153.00, 148.45, 148.38, 141.00-140.64, 139.71, 138.42, 138.04, 137.86, 136.07, 130.90-130.23, 129.09, 125.82, 124.53, 123.79, 123.43, 122.14, 120.83, 120.25, 118.33, 115.98, 112.78, 111.10, 70.62, 69.83, 69.65-69.44, 68.59, 68.09, 66.01-65.73, 64.77, 64.29, 53.66, 50.34, 47.80, 40.19, 29.80-29.50, 29.04, 28.38, 27.10, 25.84, 22.44, 21.38, 21.09, 18.31 ppm; ESI-TOF-MS (m/z): calcd for $[M+Na]^+$, $C_{154}H_{190}N_8O_{30}Na$, 2654.3486 (monoisotopic limit), 2655.3518 (most probable); found, 2654.3494

(monoisotopic limit), 2655.3534 (highest abundance peak).

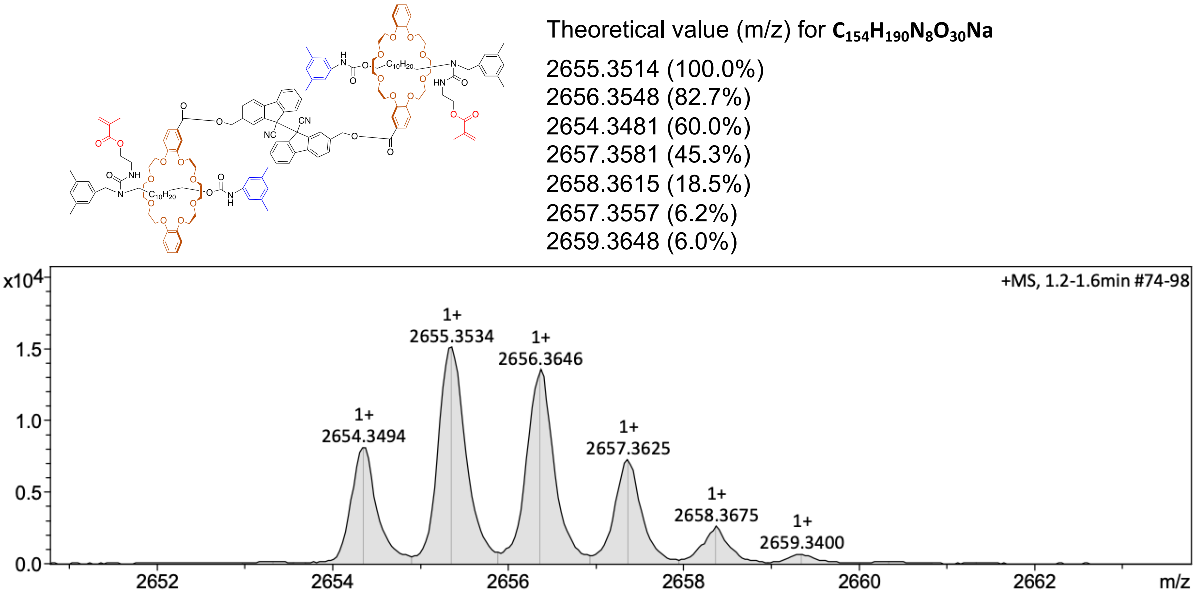

Figure S5. HR ESI-TOF MS spectrum of **CL[3]Me**.

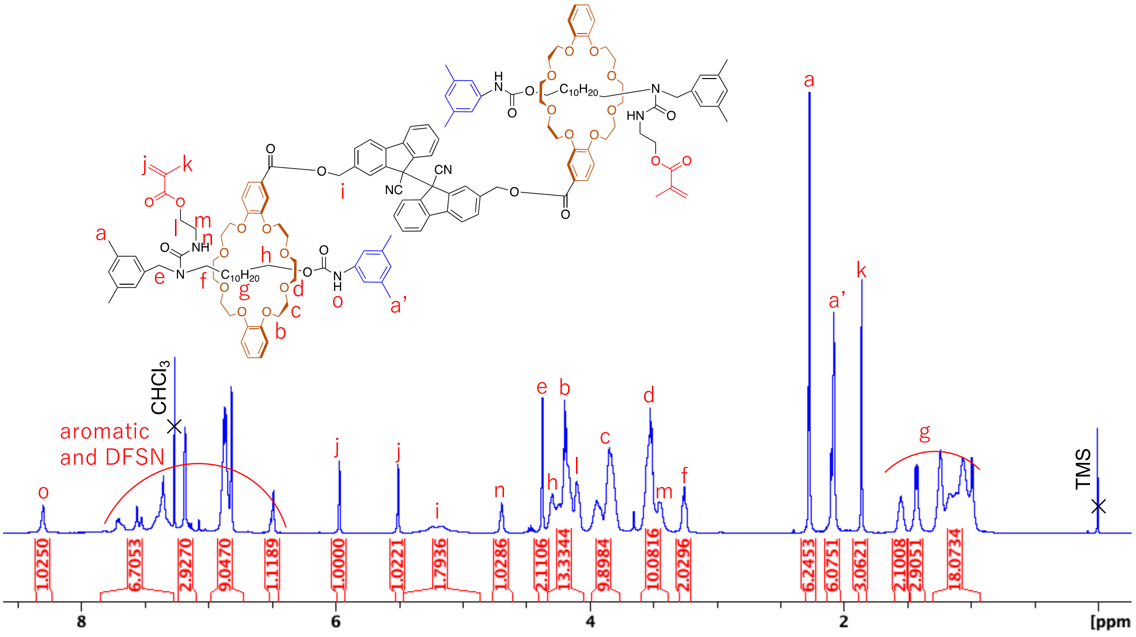

Figure S6. ^{1}H NMR spectrum of **CL[3]^{t}Bu** (500 MHz, 25 ºC, $CDCl_3$).

CL[3]^{t}Bu (299 mg, 107 µmol, 74.3%), **R = ^{t}Bu**

^{1}H-NMR (500MHz, $CDCl_3$, 298K): δ 8.14 (br, 1H), 7.70–6.80 (m, 20H), 5.96 (s, 1H), 5.50 (m, 1H), 5.19 (br, 2H), 4.75 (br, 1H), 4.41 (br, 2H), 4.36 (s, 2H), 4.27–3.50 (28H), 3.24 (t, 2H), 2.26 (s, 6H), 1.85 (s, 3H), 1.74–0.87 (m, 38H) ppm; ^{13}C-NMR (400 MHz, 298K): δ 167.44, 165.99, 158.14, 154.38, 153.02, 150.99, 148.53, 148.33, 141.00-140.64, 139.32, 138.33, 138.02, 136.09, 130.90-130.23, 128.99,

125.80, 124.58, 123.86, 122.17, 120.99, 120.30, 118.33, 115.67, 113.10, 112.78, 112.48, 111.30, 70.23-69.56, 68.83, 68.30, 66.03-65.64, 65.01, 64.29, 53.70, 50.24, 47.74, 40.14, 35.04, 34.85, 31.46, 29.89-29.55, 29.07, 28.31, 27.06, 25.87, 21.39, 18.32 ppm; ESI-TOF-MS (m/z): calcd for $[M+Na]^+$, $C_{166}H_{214}N_8O_{30}Na$, 2822.5364 (monoisotopic limit), 2823.5397 (most probable); found: 2822.5364 (monoisotopic limit), 2823.5471 (highest abundance peak), see below.

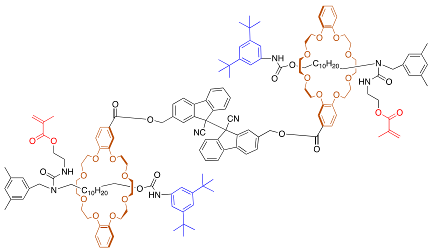

Theoretical value (m/z) for $\mathbf{C_{166}H_{214}N_8O_{30}Na}$

2823.5397 (100.0%)
2824.5431 (89.2%)
2822.5364 (55.7%)
2825.5464 (52.8%)
2826.5498 (23.3%)
2827.5531 (8.1%)
2825.5440 (6.2%)

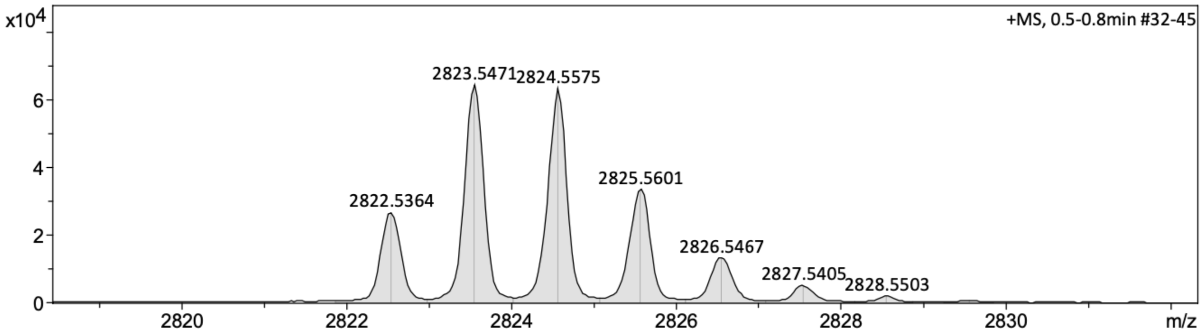

Figure S7. ESI TOF MS spectrum of **CL[3]^{t}Bu**.

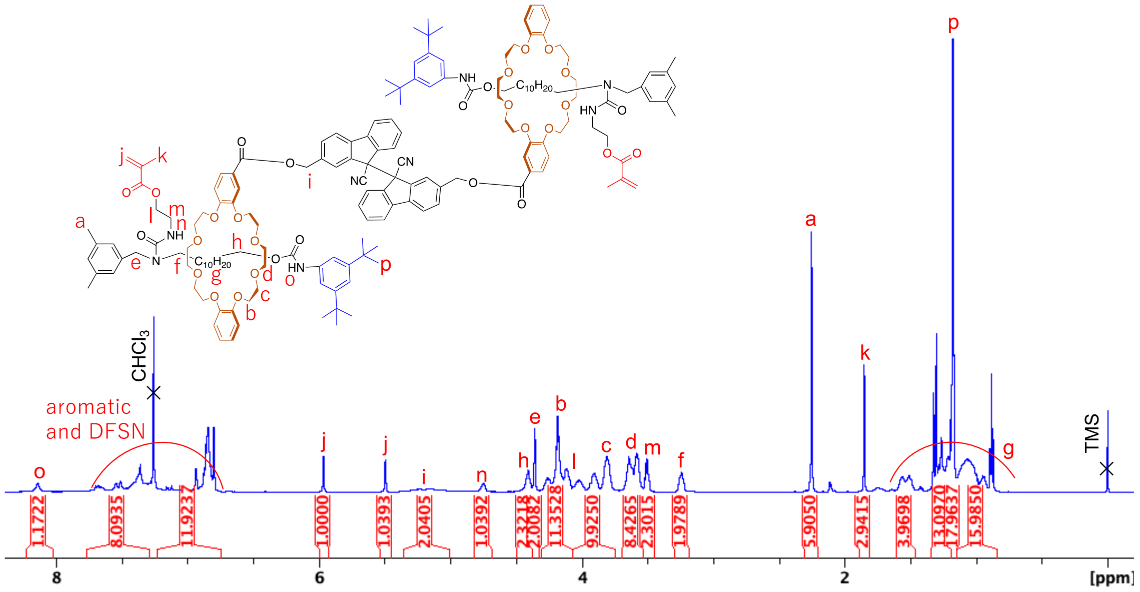

Figure S8. ^{1}H NMR spectrum of **CL[3]^{t}Bu** (500 MHz, 25 ºC, $CDCl_3$).

3. Synthesis of cross-linked polymers

General method

A solution of methyl acrylate (1011 mg, 11.7 mmol) and **crosslinker** (119 µmol) in dry DMAc (1062 µL, *ca.* 50 wt%) was degassed by freeze-pump-thaw cycling. V-70 (36.6 mg, 119 µmol) was then added, and the solution was kept under nitrogen atmosphere. After 1 minute stirring at 25 ºC ± 5 ºC, the solution was transferred via syringe onto a glass mold in a separate N_2-filled flask, which was left for 48 hours. The reaction was quenched by exposure to air, and the obtained polymer film was washed several times with a mixture of chloroform/methanol starting with v/v ratio of 3:1 and ending with 1:3. The washed film was dried at room temperature for 24 h and then in vacuo at 40 ºC to afford a colorless film of

P[2]Me, crosslinker: CL[2]Me, (1018 mg, 87.1% yield).
P[2]^{t}Bu, crosslinker: CL[2]^{t}Bu, (619 mg, 84.0% yield).
P[3]Me, crosslinker: CL[3]Me, (840 mg, 79.2% yield).
P[3]^{t}Bu, crosslinker: CL[3]^{t}Bu (831 mg, 82.2% yield).
P_{DFSN}, crosslinker: CL_{DFSN}, (759 mg, 76.7% yield).
P_{Alk}, crosslinker: CL_{Alk}, (851 mg, 80.1% yield)

4. Thermal deslipping experiments

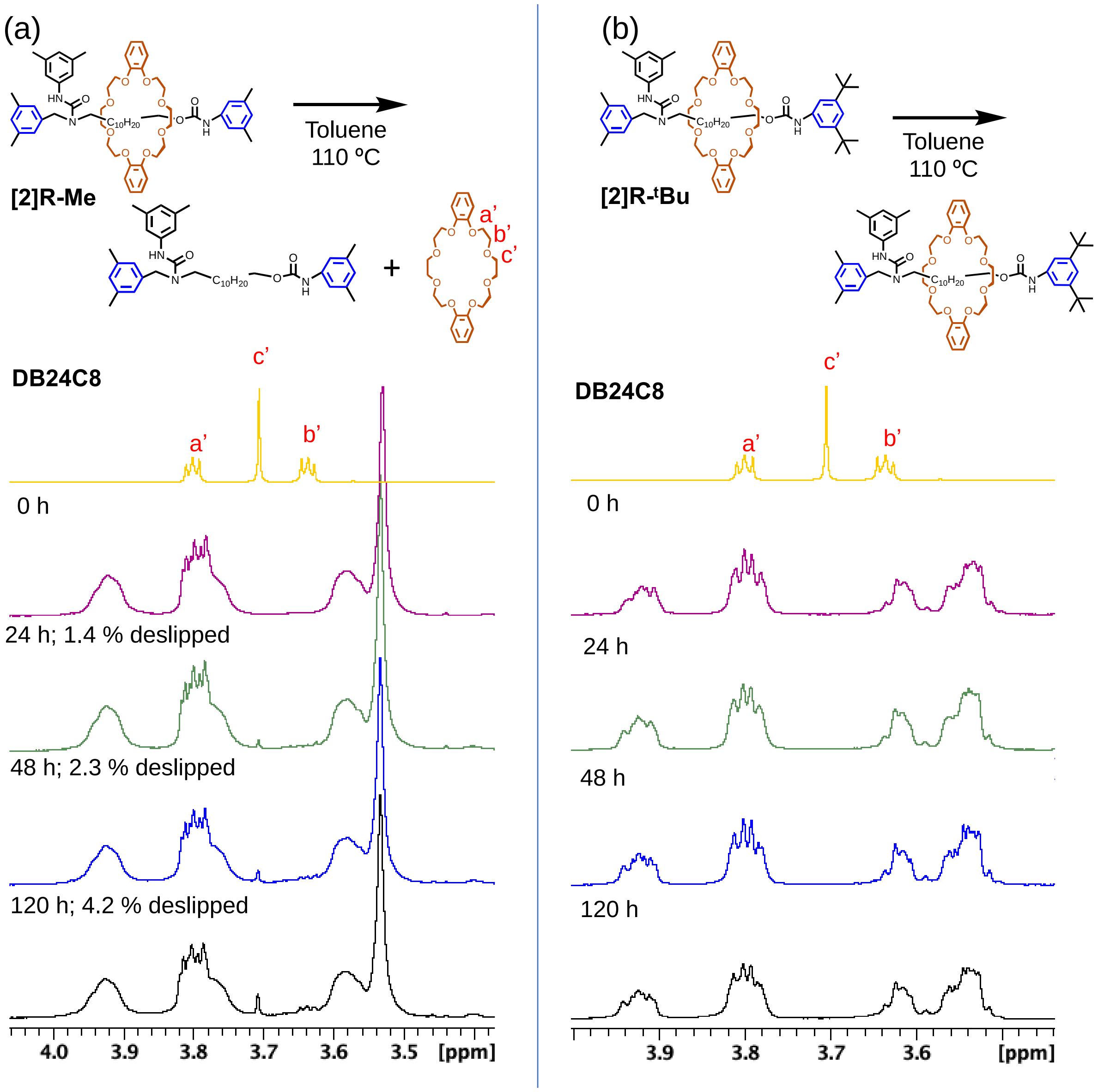

Figure S9. ^{1}H NMR spectra of (a) [2]rotaxane **[2]R-Me** and (b) [2]rotaxane **[2]R-^{t}Bu** after heated at 110 °C for 120 h. (500 MHz, 25 ºC, toluene-*δ8*).

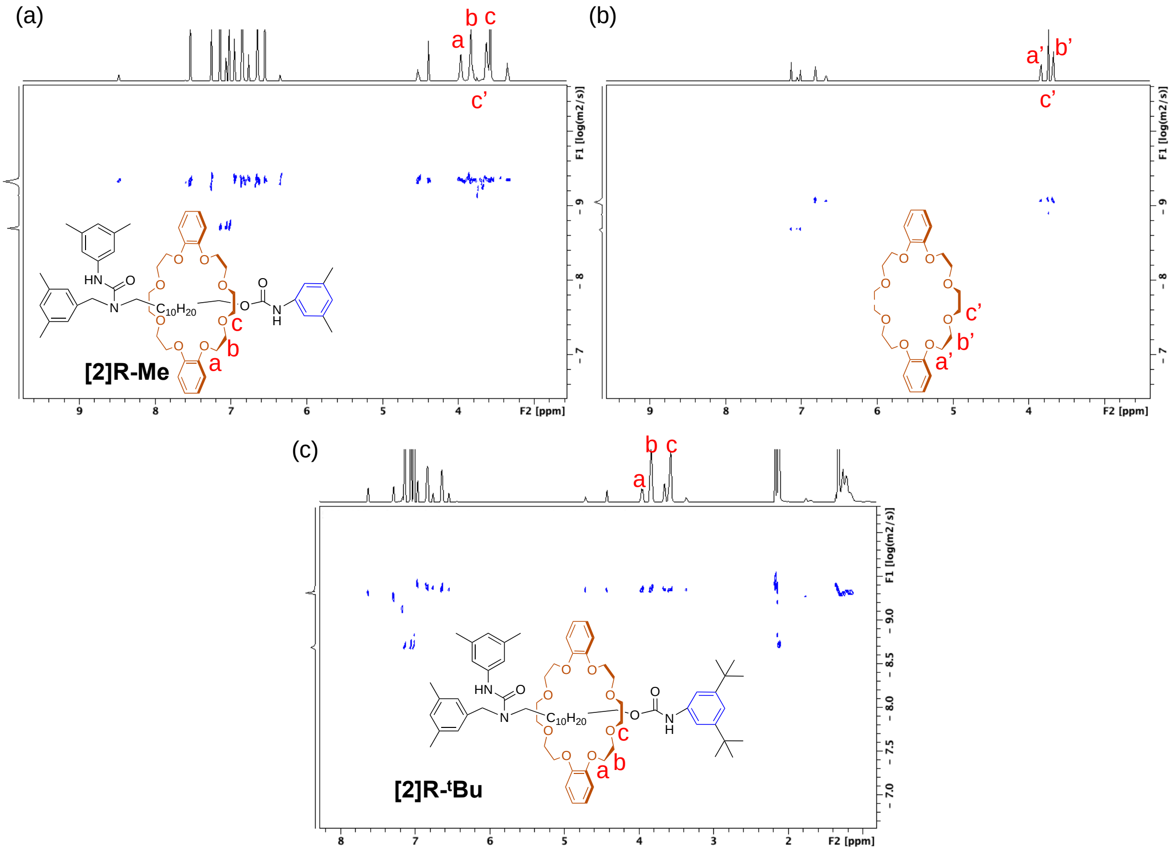

Figure S10. DOSY NMR spectrum of (a) [2]rotaxane **[2]R-Me** after heated at 110 °C for 120 h, (b) **DB24C8** and (c) [2]rotaxane **[2]R-^{t}Bu** after heated at 110 °C for 120 h. (500 MHz, 25 ºC, toluene-*δ8*).

5. Characterization of cross-linked polymers

Swelling test

In a screw-capped vial, a piece of each PMA (approx. 3 mm x 3 mm x 0.7 mm) was immersed in anisole at room temperature for 24 h. The sample was then weighed and dried before weighing again. The swelling degree Q was defined as the difference in weight of the swollen gel $W_{swollen}$ vs the dried gel W_{dry}, according to equation (1).

$$Q = \frac{W_{swallen} - W_{dry}}{W_{dry}} \quad (1)$$

Table S1. Preparation and properties of all crosslinked PMAs with various crosslinkers

Polymer	Q	T_g[a] [°C]
**P[2]Me**	5.09 ± 0.06	15.9
**P[2]^{t}Bu**	5.28 ± 0.07	16.9
**P[3]Me**	7.57 ± 0.17	22.2
**P[3]^{t}Bu**	7.68 ± 0.14	23.1
**P_{DFSN}**	5.48 ± 0.03	23.9
**P_{Alk}**	5.09 ± 0.01	20.5

[a]Estimated by DSC measurement

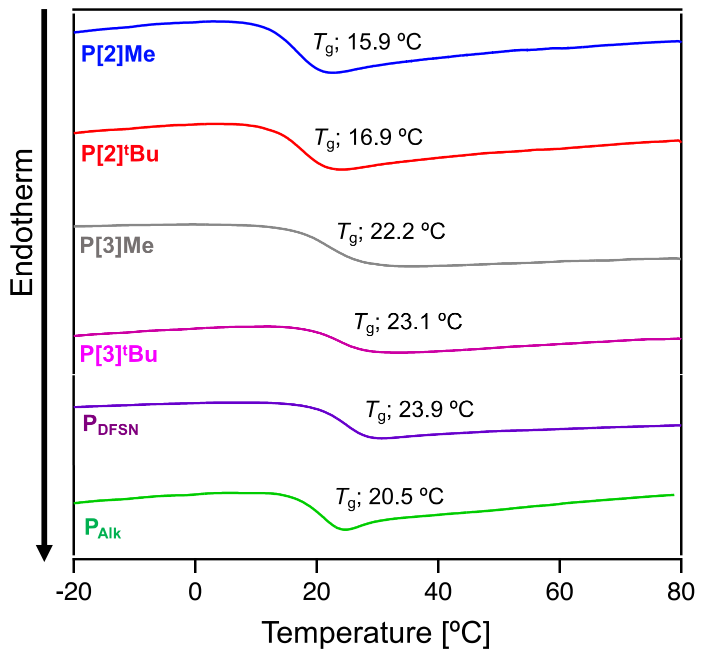

Figure S11. DSC charts of each polymer used in the study (Heating rate: 10 °C min^{-1}, 2nd heating)

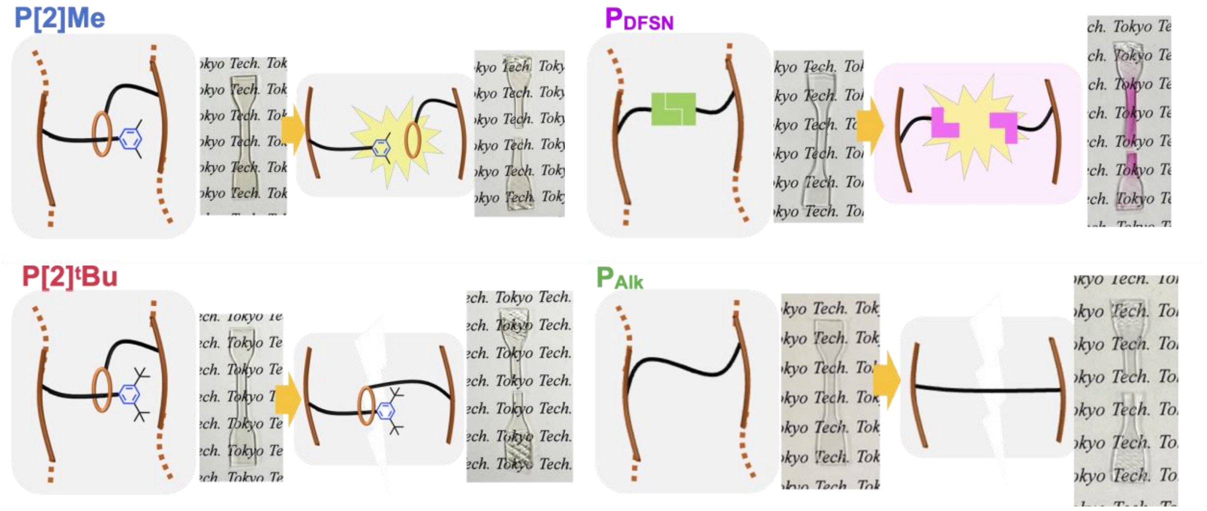

Figure S12. Photos before and after manual stretching of dumbbell-shaped elastomers containing **P[2]Me**, **P[2]^{t}Bu**, **P$_{DFSN}$** or **P$_{Alk}$**. **P[3]Me** and **P[3]^{t}Bu** are shown in the main text. Cartoons show reactions responsible for fracture/coloration.

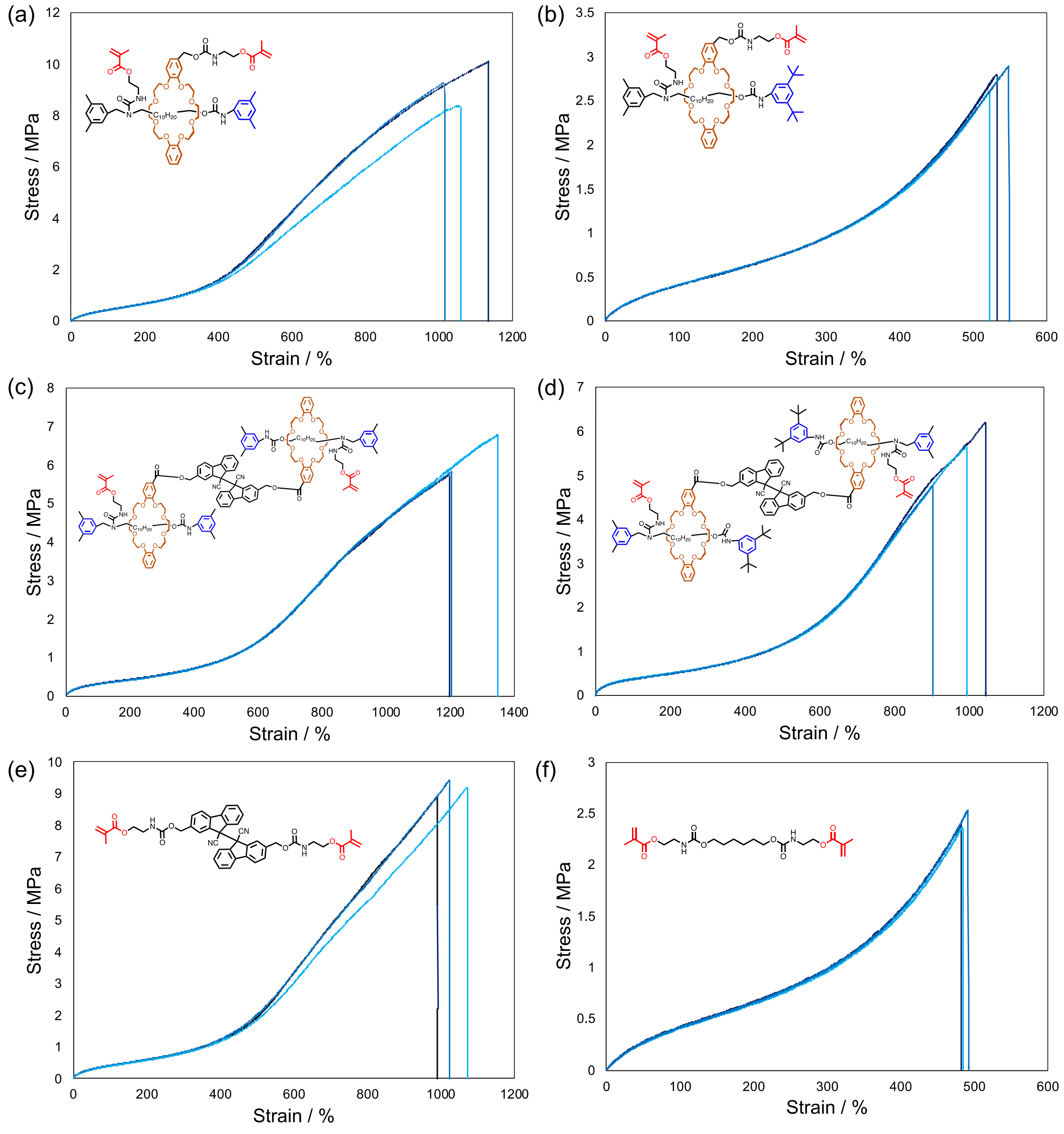

Figure S13. Stress-strain curves of (a) **P[2]Me** (sample size: 12 mm × 2 mm × 0.65 mm), (b) **P[2]^{t}Bu** (sample size: 12 mm × 2 mm × 0.66–0.70 mm), (c) **P[3]Me** (sample size: 12 mm × 2 mm × 0.70–0.73 mm), (d) **P[3]^{t}Bu** (sample size: 12 mm × 2 mm × 0.70-71 mm), (e) **P_{DFSN}** (sample size: 12 mm × 2 mm × 0.69–0.71 mm) and (f) **P_{Alk}** (sample size: 12 mm × 2 mm × 0.63–0.68 mm).

6. DFT calculations

All calculations were performed with the Gaussian 16 suite of software at the (u)BMK/6-31+G(d) level of DFT for DFSN (the UHF formalism was applied to the transition states and FSN monomer) and BLYP/6-31G(d) for rotaxanes. The Berny algorithm was used to locate stationary geometries. Tight convergence criteria and ultrafine integration grids were used in optimisations and frequency calculations (DFSN only). Conformers of DFSN were generated systematically and the strain-free conformational ensembles were built as described previously.[8] For rotaxanes, only the lowest-energy conformers of the axle and 3 lowest-energy conformers of the macrocyclic wheel were considered. The force-coupled geometries of DFSN isomers were optimized and the respective force-dependent relative energies were calculated as previously described.[8] To find f_{max}, each conformer was first optimized coupled to force of 0.5 nN (phenyl stoppers), 1.3 nN (xylyl stoppers), 2 nN (3,5-diethyl stoppers), 4 nN (3,5-isopropyl stoppers) or 5.5 nN (3,5-tBu stoppers) and than the optimizations were repeated with the coupled force increased in 50 pN (phenyl-stoppered rotaxane), 100 pN (Me and Et stoppered rotaxanes), and 250 pN (iPr-stoppered rotaxane) increments. The tBu-stoppered rotaxane was not optimized at force >5.5 nN because C-C and C-N bonds tend to homolyze on sub-s timescale at force >5.6 nN. The coupled force was implemented with iop(1/164) overlay procedure of Gaussian and used a linear harmonic constraining potential of 2×10^{-6} Hartree/Bohr^2.

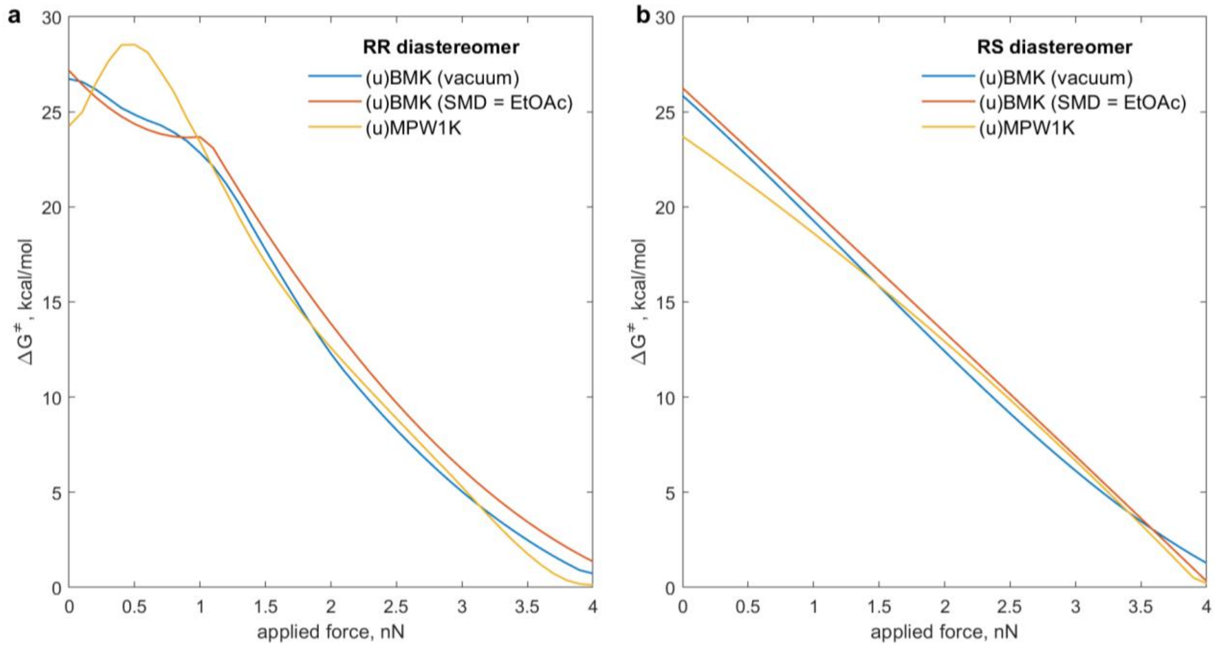

Figure S14. Force-dependent activation free energies of dissociation of the 2 diastereomers of DFSN calculated with different model chemistries at 6-31+G(d) basis set.

Table S2. Computed $\Delta G^{\ddagger}(f)$ for DFSN dissociation at (u)BMK/6-31+G(d) level in vacuum.

Force, pN	$\Delta G^{\ddagger}$, kcal/mol		Force, pN	$\Delta G^{\ddagger}$, kcal/mol	
	RR	RS		RR	RS
0	26.7	25.8	2000	12.3	12.4
100	26.6	25.2	2100	11.4	11.7
200	26.2	24.6	2200	10.6	11.1
300	25.7	24.0	2300	9.8	10.4
400	25.2	23.3	2400	9.0	9.8
500	24.9	22.6	2500	8.3	9.1
600	24.5	22.0	2600	7.6	8.5
700	24.3	21.3	2700	6.9	7.9
800	23.9	20.6	2800	6.2	7.3
900	23.5	20.0	2900	5.6	6.7
1000	22.8	19.3	3000	5.0	6.1
1100	22.1	18.6	3100	4.5	5.6
1200	21.2	17.9	3200	3.9	5.0
1300	20.2	17.2	3300	3.4	4.5
1400	18.9	16.5	3400	2.9	4.0
1500	17.7	15.8	3500	2.5	3.5
1600	16.6	15.1	3600	2.0	3.0
1700	15.5	14.4	3700	1.6	2.5
1800	14.3	13.8	3800	1.3	2.1
1900	13.3	13.1	3900	0.9	1.7
			4000	0.7	1.3

Table S3. Cartesian coordinates for converged geometries of rotaxane coupled to force closest to f_{max}.

Ph				3,5-Me2				3,5-Et2				3,5-iPr2				3,5-tBu2			
6	-3.49376	2.829747	0.463814	6	-3.21629	2.32786	1.414925	6	-3.43276	2.492454	1.028155	6	-3.91118	2.276828	0.277675	6	-3.84392	2.166447	0.375798
8	-4.01821	3.245936	1.771167	8	-4.22595	2.964461	2.278749	8	-4.2997	2.875305	2.159394	8	-4.43979	2.603406	1.660706	8	-4.40752	2.476947	1.767998
6	-3.89187	1.35791	0.210242	6	-3.76756	1.168975	0.542298	6	-3.76187	1.100802	0.424523	6	-4.50214	0.894267	-0.31723	6	-4.54534	0.884859	-0.3492
1	-2.39914	2.940897	0.425491	1	-2.43941	1.891763	2.0687	1	-2.39216	2.425183	1.382092	1	-2.81609	2.188465	0.355349	1	-2.78452	1.916467	0.543079
1	-3.94502	3.465262	-0.31307	1	-2.75885	3.098632	0.770613	1	-3.50077	3.272281	0.2525	1	-4.16605	3.103979	-0.39706	1	-3.9236	3.056796	-0.25864
8	-3.85652	1.17229	-1.2205	8	-4.26054	1.630813	-0.73763	8	-4.75424	1.132155	-0.62188	8	-5.05827	1.047781	-1.69548	8	-5.19581	1.124959	-1.69569
1	-4.89444	1.15827	0.617915	1	-4.54117	0.601105	1.086091	1	-4.07169	0.409158	1.230404	1	-5.26848	0.504078	0.369344	1	-5.29229	0.456096	0.334589
1	-3.1777	0.66566	0.705205	1	-2.90247	0.493443	0.3657	1	-2.80035	0.729675	0.006634	1	-3.67158	0.166786	-0.35957	1	-3.74064	0.145242	-0.5035
6	-4.04881	-0.17684	-1.67288	6	-4.37336	0.642203	-1.79611	6	-4.92775	-0.15047	-1.26984	6	-5.24517	-0.14798	-2.54356	6	-5.19526	0.009739	-2.69165
6	-5.50331	-0.68475	-1.81371	6	-5.77052	-0.04377	-2.00125	6	-6.36759	-0.47002	-1.7551	6	-6.63824	-0.43766	-3.32837	6	-6.49104	-0.38093	-3.61261
8	-6.11868	-0.13844	-3.00162	8	-6.31164	0.33436	-3.30956	8	-6.70965	0.083899	-3.05787	8	-6.83074	0.127028	-4.69227	8	-6.77955	0.370483	-4.86542
1	-6.09628	-0.44785	-0.91052	1	-6.45967	0.252274	-1.19325	1	-7.08321	-0.15038	-0.97728	1	-7.48989	-0.17405	-2.67672	1	-7.39289	-0.41896	-2.97834
1	-5.45965	-1.79336	-1.90434	1	-5.66359	-1.14853	-1.97866	1	-6.42664	-1.57762	-1.83069	1	-6.63938	-1.53958	-3.44352	1	-6.24279	-1.41961	-3.91096
6	-7.43222	-0.66578	-3.25528	6	-7.59362	-0.26837	-3.60774	6	-7.97184	-0.42447	-3.54661	6	-8.2244	0.172018	-5.18708	6	-7.97992	-0.00044	-5.66436
6	-7.97239	-0.13642	-4.59808	6	-8.0824	0.033605	-5.05706	6	-8.28302	-0.05583	-5.0276	6	-8.33835	0.849257	-6.63549	6	-8.25553	1.051867	-6.86365
1	-7.37909	-1.77442	-3.32017	1	-7.49772	-1.37061	-3.51195	1	-7.94945	-1.53472	-3.49595	1	-8.63385	-0.85496	-5.2346	1	-7.81616	-1.00332	-6.10184
1	-8.13911	-0.4145	-2.43933	1	-8.3632	0.061882	-2.882	1	-8.81455	-0.08764	-2.90884	1	-8.85335	0.755488	-4.49116	1	-8.87273	-0.04918	-5.01853
6	-3.49806	4.395901	2.351338	6	-3.84518	4.063674	3.042631	6	-3.92552	3.956999	2.959285	6	-4.17271	3.772714	2.407364	6	-4.27074	3.61209	2.624834
8	-8.75214	1.07309	-4.40678	8	-9.04795	1.134081	-5.08738	8	-9.05337	1.176305	-5.13376	8	-9.64302	1.56042	-6.76142	8	-9.72661	1.330894	-7.06517
1	-8.62024	-0.90308	-5.06334	1	-8.57905	-0.87378	-5.44459	1	-8.88194	-0.88368	-5.44863	1	-8.25148	0.079586	-7.4188	1	-7.83882	0.657054	-7.8033
1	-7.11428	0.050186	-5.26903	1	-7.2214	0.275288	-5.70591	1	-7.34457	0.028549	-5.60442	1	-7.52269	1.579873	-6.7615	1	-7.76368	2.00744	-6.62799
6	-9.3687	1.630912	-5.52878	6	-9.69925	1.475943	-6.27477	6	-9.67075	1.535753	-6.33714	6	-10.2226	2.043764	-7.97406	6	-10.3071	1.928515	-8.24294
6	-10.3318	2.672716	-5.32648	6	-10.4921	2.674263	-6.27687	6	-10.3692	2.789638	-6.36635	6	-11.0468	3.230372	-7.91825	6	-11.1952	3.067631	-8.14758
8	-10.7869	3.151048	-4.10539	8	-10.5142	3.607773	-5.23359	8	-10.3534	3.729759	-5.33252	8	-11.1295	4.11338	-6.80667	8	-11.3876	3.902059	-7.00101
1	-3.51438	-0.8832	-1.00257	1	-3.59827	-0.13548	-1.64559	1	-4.69064	-0.95508	-0.54731	1	-5.12941	-1.04712	-1.91194	1	-5.03022	-0.93321	-2.13904
1	-3.57355	-0.22354	-2.6676	1	-4.13599	1.187975	-2.72406	1	-4.22186	-0.24347	-2.12136	1	-4.43938	-0.15837	-3.30058	1	-4.33813	0.165907	-3.37257
6	-4.38636	5.328457	2.984375	6	-4.84587	4.969164	3.568063	6	-4.91933	4.828949	3.549384	6	-5.16091	4.683031	3.082961	6	-5.33582	4.558233	3.152527
6	-3.85752	6.465927	3.624132	6	-4.43601	6.068811	4.346337	6	-4.50205	5.88241	4.386553	6	-4.63816	5.716046	3.885025	6	-4.93011	5.48092	4.133774
6	-2.47118	6.703472	3.651796	6	-3.07879	6.317619	4.624683	6	-3.14515	6.116234	4.66371	6	-3.26577	5.957705	4.058187	6	-3.61463	5.59207	4.621067
6	-1.5977	5.792973	3.044534	6	-2.10464	5.449072	4.116014	6	-2.17739	5.27487	4.103085	6	-2.34443	5.124404	3.4163	6	-2.62749	4.735432	4.12216
6	-2.11391	4.650339	2.409033	6	-2.49096	4.341957	3.33679	6	-2.5726	4.21417	3.271119	6	-2.80877	4.06204	2.625377	6	-2.97467	3.774868	3.15481
6	-9.09893	1.215032	-6.85206	6	-9.62585	0.730765	-7.47548	6	-9.64828	0.766265	-7.5223	6	-10.1501	1.398166	-9.2363	6	-10.2071	1.335283	-9.53407
6	-10.9799	3.224971	-6.45612	6	-11.1606	3.05347	-7.47027	6	-11.0018	3.193054	-7.57266	6	-11.7293	3.64573	-9.12826	6	-11.9007	3.483753	-9.35779
6	-10.7199	2.787898	-7.77113	6	-11.0777	2.309737	-8.67869	6	-10.9718	2.424299	-8.76849	6	-11.656	2.995969	-10.4254	6	-11.7973	2.883239	-10.6837
6	-9.76377	1.777958	-7.9597	6	-10.2974	1.137472	-8.65444	6	-10.2824	1.198469	-8.71242	6	-10.8222	1.849613	-10.4119	6	-10.8961	1.783154	-10.7024
1	-0.51696	5.957899	3.065615	1	-1.04268	5.622054	4.31338	1	-1.11447	5.431306	4.306757	1	-1.26814	5.280223	3.533268	1	-1.59528	4.794082	4.480621
1	-1.42735	3.932096	1.95607	1	-1.71552	3.682397	2.945638	1	-1.80173	3.563948	2.85519	1	-2.08461	3.394697	2.150924	1	-2.2111	3.084254	2.784929
1	-4.52898	7.180753	4.101666	1	-5.1948	6.745798	4.744049	1	-5.25833	6.532223	4.830703	1	-5.36736	6.354069	4.390431	1	-5.70716	6.141337	4.528208
1	-11.7144	4.010731	-6.25946	1	-11.7353	3.983927	-7.42918	1	-11.5104	4.161213	-7.54564	1	-12.3304	4.554057	-9.01403	1	-12.558	4.348647	-9.21455
1	-11.248	3.237721	-8.61549	1	-11.5999	2.641788	-9.57863	1	-11.4654	2.776319	-9.67612	1	-12.1903	3.369914	-11.2975	1	-12.3577	3.256103	-11.54
1	-9.52439	1.414304	-8.96319	1	-10.1912	0.521209	-9.55291	1	-10.2172	0.556795	-9.59651	1	-10.675	1.261447	-11.3241	1	-10.7185	1.226503	-11.6292
1	-8.36338	0.429068	-7.02819	1	-9.01835	-0.17626	-7.50907	1	-9.10645	-0.18192	-7.53586	1	-9.54032	0.493432	-9.31935	1	-9.56369	0.454605	-9.63321
8	-5.75447	5.060771	2.944978	8	-6.20535	4.739557	3.320151	8	-6.28076	4.617873	3.307192	8	-6.62164	4.648562	3.128048	8	-6.78026	4.626249	2.921259
6	-6.69279	6.065887	3.413511	6	-7.16022	5.858201	3.282363	6	-7.23351	5.725807	3.385059	6	-7.55482	5.664831	2.431261	6	-7.61935	5.624604	2.092546

Ph				3,5-Me2				3,5-Et2				3,5-iPr2				3,5-tBu2			
6	-8.13339	5.577999	3.197818	6	-8.53638	5.406814	2.726404	6	-8.59312	5.333684	2.751089	6	-8.8388	5.102815	1.600016	6	-8.92948	4.974066	1.386602
1	-6.52812	7.017274	2.876382	1	-6.74509	6.674766	2.666714	1	-6.81931	6.61688	2.882145	1	-6.96621	6.358652	1.820506	1	-6.99622	6.122222	1.346522
1	-6.53978	6.235541	4.495482	1	-7.31176	6.218024	4.315173	1	-7.41167	5.966235	4.448766	1	-7.96336	6.211015	3.293638	1	-7.95789	6.353746	2.843516
8	-8.50554	5.678299	1.816349	8	-8.61025	5.54436	1.291776	8	-8.59911	5.540452	1.328777	8	-8.93257	5.504218	0.182068	8	-9.19634	5.438371	-0.00984
1	-8.79665	6.216423	3.823184	1	-9.30597	6.055916	3.196798	1	-9.36876	5.969333	3.231584	1	-9.72306	5.52167	2.121414	1	-9.80605	5.259933	1.998734
1	-8.22914	4.537632	3.568304	1	-8.74205	4.365066	3.03776	1	-8.82458	4.28229	3.00985	1	-8.89456	4.006612	1.693726	1	-8.85401	3.877932	1.391131
6	-9.81773	5.12992	1.559799	6	-9.71042	4.816032	0.668523	6	-9.65302	4.830465	0.617401	6	-9.95352	4.776444	-0.61497	6	-10.2356	4.635018	-0.72819
6	-10.2748	5.374801	0.108652	6	-9.77817	5.234304	-0.82379	6	-9.61675	5.279942	-0.86658	6	-10.0853	5.382658	-2.08182	6	-10.7367	5.286094	-2.11135
1	-10.5492	5.620773	2.237208	1	-10.6604	5.051765	1.188524	1	-10.6391	5.052535	1.072452	1	-10.9349	4.835777	-0.10556	1	-11.108	4.521369	-0.05815
1	-9.83065	4.042633	1.76837	1	-9.52423	3.729603	0.742409	1	-9.47643	3.741488	0.673954	1	-9.66142	3.716143	-0.69543	1	-9.82176	3.636475	-0.9356
8	-9.85335	4.319489	-0.76726	8	-10.4433	4.193918	-1.59661	8	-10.2816	4.285116	-1.68559	8	-10.811	4.458591	-2.99169	8	-10.9865	4.245686	-3.15833
1	-9.89679	6.359981	-0.23633	1	-8.74872	5.374116	-1.19864	1	-8.56248	5.383299	-1.17798	1	-9.07036	5.565253	-2.4714	1	-9.98123	6.010198	-2.46273
1	-11.3855	5.428595	0.115556	1	-10.3277	6.191229	-0.9332	1	-10.1102	6.267263	-0.98134	1	-10.6219	6.349199	-2.0373	1	-11.6805	5.834767	-1.93919
6	-10.4948	4.38021	-2.05573	6	-10.5428	4.531729	-3.00513	6	-10.2683	4.630721	-3.0897	6	-10.9128	4.900364	-4.40725	6	-11.3259	4.690824	-4.54481
6	-9.91537	3.256678	-2.94149	6	-10.8763	3.26823	-3.8497	6	-10.6361	3.38195	-3.93503	6	-11.2915	3.691165	-5.37693	6	-11.3104	3.447874	-5.55951
1	-10.3299	5.366568	-2.53621	1	-9.57929	4.935261	-3.36353	1	-9.26029	4.968132	-3.39403	1	-9.95132	5.321615	-4.74554	1	-10.5916	5.441703	-4.88485
1	-11.592	4.238659	-1.95028	1	-11.3171	5.311885	-3.15154	1	-10.976	5.460729	-3.29507	1	-11.6804	5.693048	-4.48278	1	-12.3288	5.155822	-4.53634
1	-9.90629	2.307543	-2.3833	1	-11.9531	3.022348	-3.81291	1	-11.7068	3.127404	-3.83074	1	-12.3465	3.405077	-5.2249	1	-12.1826	2.800742	-5.37065
1	-8.88827	3.494335	-3.24389	1	-10.2958	2.40755	-3.49309	1	-10.03	2.528382	-3.60989	1	-10.6502	2.829369	-5.17201	1	-10.3969	2.866871	-5.42121
6	-6.81291	3.274236	0.111659	6	-6.64866	3.261831	0.300593	6	-6.67392	3.245712	0.211712	6	-6.81602	2.889731	0.597992	6	-6.66413	3.005443	0.541323
6	-6.96897	2.387904	-0.96811	6	-7.18348	2.862032	-0.93898	6	-7.04277	2.82164	-1.08329	6	-7.22115	2.40155	-0.67688	6	-7.29261	2.680651	-0.69275
6	-6.6471	2.815138	-2.2706	6	-6.81934	3.553717	-2.12543	6	-6.62961	3.557492	-2.22607	6	-6.96764	3.179036	-1.87078	6	-7.11879	3.52396	-1.85843
6	-6.21938	4.125216	-2.52958	6	-5.94609	4.653801	-2.06338	6	-5.90383	4.753086	-2.06772	6	-6.44787	4.473192	-1.8075	6	-6.38149	4.70218	-1.77627
6	-6.11323	5.026614	-1.45697	6	-5.43532	5.100621	-0.83598	6	-5.5715	5.229897	-0.79355	6	-6.03745	4.993911	-0.5926	6	-5.69975	5.052188	-0.62306
1	-5.97111	4.434944	-3.54911	6	-7.34607	3.172098	-3.49115	6	-6.93853	3.129028	-3.65446	6	-7.06231	2.698644	-3.32154	6	-7.52218	3.232539	-3.3293
1	-7.31354	1.372105	-0.78423	6	-4.52712	6.320954	-0.75983	6	-4.87061	6.570661	-0.60013	6	-5.36092	6.367398	-0.4792	6	-4.78737	6.320025	-0.6526
6	-6.39897	4.606187	-0.15382	1	-5.68418	5.177725	-2.99095	1	-5.60986	5.322094	-2.95712	1	-6.29431	5.036331	-2.7296	1	-6.29037	5.332215	-2.65749
1	-6.29649	5.307326	0.66826	1	-7.90133	2.039558	-0.98727	1	-7.63811	1.918845	-1.21566	1	-7.65669	1.403305	-0.77613	1	-7.89237	1.770612	-0.76317
7	-7.01938	2.93052	1.482708	6	-5.80147	4.399195	0.31961	6	-5.95658	4.462389	0.315501	6	-6.22296	4.179465	0.522055	6	-5.86976	4.195257	0.470175
6	-6.98697	1.692917	2.103555	1	-5.42434	4.768863	1.26085	1	-5.70022	4.844545	1.291516	1	-5.80177	4.624659	1.378042	1	-5.29635	4.538695	1.29308
8	-6.89352	1.925318	3.479155	7	-7.01537	2.714108	1.604511	7	-7.08524	2.641528	1.487359	7	-7.05498	2.269559	2.016435	7	-6.99744	2.243619	1.943045
6	-6.63165	0.781581	4.355155	6	-6.80372	1.471594	2.203689	6	-6.72551	1.438628	2.131656	6	-6.2809	1.359165	2.867791	6	-6.24254	1.348708	2.872725
6	-6.17086	1.351913	5.717621	8	-6.75161	1.686048	3.600341	8	-6.67058	1.708456	3.52275	8	-6.11659	1.875441	4.241014	8	-6.09112	1.881047	4.265017
1	-5.86142	0.142439	3.89263	6	-6.30245	0.61313	4.513546	6	-6.1403	0.682037	4.44387	6	-5.4093	0.977303	5.32953	6	-5.38413	0.97413	5.454857
1	-7.55404	0.181882	4.453578	6	-5.92955	1.261876	5.890005	6	-5.84396	1.343577	5.838858	6	-5.1224	1.669851	6.814582	6	-5.04777	1.686621	6.94866
6	-5.8371	0.284197	6.803915	1	-5.43763	0.103151	4.057871	1	-5.2242	0.255198	4.003308	1	-4.46109	0.65786	4.875384	1	-4.45923	0.618751	4.984609
1	-6.9634	2.023319	6.097179	1	-7.1199	-0.12113	4.621759	1	-6.88155	-0.131	4.538893	1	-6.05575	0.096205	5.452368	1	-6.08649	0.141158	5.584972
1	-5.28415	1.986633	5.538964	6	-5.42625	0.270428	7.011832	6	-5.25549	0.385522	6.956029	6	-4.37402	0.763075	8.041567	6	-4.31192	0.76878	8.242087
8	-7.04173	0.573751	1.589505	1	-6.81801	1.804823	6.262652	1	-6.78284	1.794462	6.209119	1	-6.09326	2.017445	7.205298	1	-5.99945	2.079527	7.34172
1	-6.68728	3.665496	2.110803	1	-5.15119	2.02394	5.705447	1	-5.14133	2.179195	5.672466	1	-4.50695	2.56788	6.640777	1	-4.38877	2.547848	6.755416
6	-5.39136	0.913552	8.160139	8	-6.70314	0.364832	1.670382	8	-6.54571	0.325254	1.648717	8	-5.88824	0.246056	2.552473	8	-5.8866	0.213218	2.597461
1	-5.03753	-0.38401	6.430403	1	-6.82547	3.425505	2.316443	1	-6.98952	3.367264	2.200001	1	-7.22275	3.08834	2.610984	1	-7.30641	3.023534	2.532833
1	-6.72196	-0.35904	6.974531	6	-5.0621	0.963902	8.384238	6	-4.95953	1.072643	8.353786	6	-5.68785	2.816327	-4.05732	6	-6.19221	2.931773	-4.10997
6	-5.05107	-0.10055	9.295527	1	-4.53639	-0.27434	6.640521	1	-4.3188	-0.06822	6.579575	1	-5.72294	2.255065	-5.00201	1	-6.39112	2.73998	-5.17297
1	-6.19363	1.588681	8.516935	1	-6.20457	-0.49576	7.195088	1	-5.95917	-0.45366	7.116396	1	-4.8618	2.429504	-3.44754	1	-5.62079	2.100394	-3.70442
1	-4.51007	1.559272	7.980784	6	-4.55981	0.018423	9.547042	6	-4.37273	0.145606	9.499588	1	-5.4662	3.869096	-4.30246	1	-5.54459	3.821824	-4.04806

Ph				3,5-Me2				3,5-Et2				3,5-iPr2				3,5-tBu2			
6	-4.64872	0.588736	10.6356	1	-5.95365	1.51449	8.743555	1	-5.8981	1.529272	8.722916	6	-8.10412	3.480186	-4.13857	6	-8.61958	2.151482	-3.36472
1	-4.23188	-0.7654	8.959651	1	-4.2858	1.73039	8.194084	1	-4.25826	1.912714	8.188557	1	-7.84837	4.552634	-4.18409	1	-8.91059	1.913372	-4.39119
1	-5.92661	-0.75573	9.470294	6	-4.2301	0.763758	10.89947	6	-4.11838	0.869895	10.88535	1	-9.09613	3.382526	-3.68677	1	-9.52073	2.524652	-2.86066
6	-4.36487	-0.38839	11.81724	1	-3.6602	-0.52792	9.202104	1	-3.42549	-0.30365	9.144672	1	-8.14793	3.12416	-5.17879	1	-8.31208	1.226269	-2.86634
1	-5.4613	1.282764	10.9176	1	-5.33412	-0.74983	9.739356	1	-5.07026	-0.69859	9.661806	6	-6.2627	7.50508	-1.03149	6	-8.05656	4.500215	-4.08541
1	-3.74893	1.211711	10.48034	6	-3.79696	-0.14344	12.11973	6	-3.61397	-0.03252	12.09117	1	-6.40629	7.403998	-2.12154	1	-7.24399	5.187111	-4.37012
7	-3.93781	0.295324	13.06945	1	-5.12685	1.343481	11.18351	1	-5.0654	1.357132	11.17872	1	-5.79921	8.490744	-0.85003	1	-8.78369	5.067462	-3.50634
1	-3.54505	-1.06927	11.52531	1	-3.42528	1.501616	10.72405	1	-3.38456	1.684075	10.7443	1	-7.25941	7.487494	-0.56331	1	-8.52907	4.182168	-5.0281
1	-5.265	-1.03013	11.98855	7	-3.50757	0.61341	13.38784	7	-3.41146	0.711507	13.38634	6	-3.96954	6.385228	-1.17094	6	-5.639	7.56928	-1.04973
6	-5.02292	1.081642	13.67693	1	-2.87173	-0.68376	11.85142	1	-2.63872	-0.47412	11.81977	1	-4.05623	6.167787	-2.24945	1	-6.12089	7.448233	-2.03343
1	-5.37902	1.855413	12.9799	1	-4.58697	-0.9159	12.29336	1	-4.32695	-0.88293	12.22089	1	-3.29348	5.634807	-0.72807	1	-4.9954	8.466069	-1.09948
1	-4.64204	1.593394	14.57664	6	-4.72033	1.237351	13.94702	6	-4.68413	1.20068	13.94003	1	-3.49396	7.376101	-1.06441	1	-6.43665	7.764113	-0.31242
6	-3.36912	-0.63157	14.07865	1	-5.17216	1.93147	13.2225	1	-5.1937	1.861212	13.22207	6	-4.05933	1.413588	9.575074	6	-4.07604	6.64054	0.688043
1	-2.08885	7.598131	4.150691	1	-4.45067	1.820439	14.84361	1	-4.484	1.792362	14.84941	1	-3.40847	0.414157	7.63625	1	-4.7846	6.857088	1.50271
1	-5.90143	0.461204	13.9823	6	-2.81651	-0.16996	14.47206	6	-2.65954	-0.01704	14.47481	1	-4.99349	-0.13746	8.195503	1	-3.44617	7.537985	0.557727
1	-4.10622	-1.37366	14.46895	1	-2.79963	7.184273	5.230455	1	-2.85999	6.947649	5.313832	6	-3.31207	0.50132	10.79739	1	-3.41773	5.822803	1.021317
1	-2.98994	-0.04477	14.93133	1	-5.49688	0.488051	14.2434	1	-5.3936	0.381821	14.21668	1	-5.0247	1.763028	9.980712	6	-3.67289	6.114973	-1.73094
1	-2.52528	-1.18657	13.63596	1	-3.45267	-0.98622	14.88887	1	-3.21156	-0.89454	14.88327	1	-3.43925	2.313717	9.421635	1	-4.09375	5.935716	-2.7336
1	-6.72507	2.100321	-3.08533	1	-2.55168	0.524221	15.28491	1	-2.47272	0.69418	15.2944	6	-3.00872	1.161449	12.326	1	-3.03013	5.253433	-1.47844
1	-5.79144	6.059402	-1.62627	1	-1.89154	-0.6143	14.06915	1	-1.69168	-0.3657	14.07902	1	-2.34664	0.151758	10.39218	1	-3.0298	7.011701	-1.79039
				1	-8.20449	2.500701	-3.42878	1	-7.62675	2.281214	-3.65419	1	-3.93316	-0.39873	10.94935	6	-3.94244	1.439763	9.800005
				1	-5.0559	7.237181	-1.0828	1	-4.11642	6.717654	-1.39524	6	-2.32163	0.256843	13.60985	1	-3.36457	0.374086	7.838029
				1	-3.64692	6.20975	-1.41934	1	-7.45927	3.95088	-4.18015	1	-3.97704	1.540992	12.69125	1	-4.974	-0.09484	8.421762
				1	-7.6521	4.063453	-4.06435	1	-4.3204	6.566631	0.357977	1	-2.35646	2.040192	12.18968	6	-3.20801	0.519776	11.085
				1	-4.15874	6.49108	0.265352	6	-5.67596	2.763112	-4.47634	7	-2.15112	0.964127	15.01476	1	-4.89034	1.83455	10.20317
				1	-6.57237	2.653768	-4.07974	1	-5.14131	1.919718	-4.01548	1	-1.31272	-0.05128	13.29419	1	-3.28138	2.304156	9.619612
								1	-4.97957	3.615984	-4.54929	1	-2.94147	-0.65919	13.71287	6	-2.84681	1.196781	12.63871
								1	-5.95826	2.474513	-5.50434	6	-3.48207	1.310973	15.53861	1	-2.26043	0.125457	10.68053
								6	-5.85654	7.769237	-0.61293	1	-4.03698	1.91787	14.80498	1	-3.86966	-0.34506	11.26256
								1	-6.63816	7.640882	0.154625	1	-3.37696	1.920726	16.45335	6	-2.14752	0.289001	13.98373
								1	-5.32919	8.720359	-0.42215	6	-1.32126	0.349847	16.29822	1	-3.79598	1.611337	13.01389
								1	-6.3636	7.85121	-1.58945	1	-2.93485	6.784788	4.692078	1	-2.16158	2.044484	12.47449
												1	-4.12015	0.428923	15.79635	7	-1.9368	1.021007	15.40568
												1	-1.84339	-0.51338	16.75046	1	-1.14922	-0.04054	13.66077
												1	-1.26761	1.191658	16.99908	1	-2.79422	-0.60203	14.11758
												1	-0.31749	0.060851	15.95728	6	-3.26744	1.386026	15.92811
												1	-5.18292	6.571283	0.593093	1	-3.81096	1.994592	15.18615
												1	-7.37489	1.661246	-3.31553	1	-3.15853	2.000557	16.83911
																6	-1.09446	0.403663	16.81414
																1	-3.38072	6.341791	5.382655
																1	-3.91362	0.509242	16.18532
																1	-1.68305	-0.40618	17.27433
																1	-1.04234	1.304651	17.43318
																1	-0.10434	0.066985	16.48329

7. SI references

[1] H. Sakai, T. Sumi, D. Aoki, R. Goseki, H. Otsuka, *ACS Macro Lett.* **2018**, *7*, 1359–1363.

[2] H. Sakai, D. Aoki, K. Seshimo, K. Mayumi, S. Nishitsuji, T. Kurose, H. Ito, H. Otsuka, *ACS Macro Lett.* **2020**, *9*, 1108–1113.

[3] K. Iijima, Y. Kohsaka, Y. Koyama, K. Nakazono, S. Uchida, S. Asai, T. Takata, *Polym. J.* **2014**, *46*, 67–72.

[4] D. Feng, X. Li, X. Wang, X. Jiang, Z. Li, *Tetrahedron* **2004**, *60*, 6137–6144.

[5] D. Aoki, S. Uchida, K. Nakazono, Y. Koyama, T. Takata, *ACS Macro Lett.* **2013**, 461–465.

[6] Y. H. Lin, C. C. Lai, Y. H. Liu, S. M. Peng, S. H. Chiu, *Angew. Chem., Int. Ed.* **2013**, *52*, 10231–10236.

[7] J. Sawada, D. Aoki, T. Takata, *Macromol. Symp.* **2017**, *372*, 115–119.

[8] S. Akbulatov, Y. Tian, Z. Huang, T. J. Kucharski, Q. Z. Yang, R. Boulatov, *Science* **2017**, *357*, 299–303.
